# Genomic Stability and Genetic Defense Systems in *Dolosigranulum pigrum*, a Candidate Beneficial Bacterium from the Human Microbiome

**DOI:** 10.1128/mSystems.00425-21

**Published:** 2021-09-21

**Authors:** Stephany Flores Ramos, Silvio D. Brugger, Isabel Fernandez Escapa, Chelsey A. Skeete, Sean L. Cotton, Sara M. Eslami, Wei Gao, Lindsey Bomar, Tommy H. Tran, Dakota S. Jones, Samuel Minot, Richard J. Roberts, Christopher D. Johnston, Katherine P. Lemon

**Affiliations:** a The Forsyth Institute (Microbiology), Cambridge, Massachusetts, USA; b Department of Infectious Diseases and Hospital Epidemiology, University Hospital Zurich, University of Zurich, Zurich, Switzerland; c Department of Oral Medicine, Infection, and Immunity, Harvard School of Dental Medicine, Boston, Massachusetts, USA; d Alkek Center for Metagenomics and Microbiome Research, Department of Molecular Virology and Microbiology, Baylor College of Medicine, Houston, Texas, USA; e Vaccine and Infectious Diseases Division, Fred Hutchinson Cancer Research Center, Seattle, Washington, USA; f New England Biolabs, Ipswich, Massachusetts, USA; g Division of Infectious Diseases, Boston Children’s Hospital, Harvard Medical School, Boston, Massachusetts, USA; h Section of Infectious Diseases, Texas Children’s Hospital, Department of Pediatrics, Baylor College of Medicine, Houston, Texas, USA; University of Trento

**Keywords:** *Dolosigranulum pigrum*, nasal microbiota, pangenome, methylome, restriction modification, CRISPR, mobile genetic elements

## Abstract

Dolosigranulum pigrum is positively associated with indicators of health in multiple epidemiological studies of human nasal microbiota. Knowledge of the basic biology of *D. pigrum* is a prerequisite for evaluating its potential for future therapeutic use; however, such data are very limited. To gain insight into *D. pigrum*’s chromosomal structure, pangenome, and genomic stability, we compared the genomes of 28 *D. pigrum* strains that were collected across 20 years. Phylogenomic analysis showed closely related strains circulating over this period and closure of 19 genomes revealed highly conserved chromosomal synteny. Gene clusters involved in the mobilome and in defense against mobile genetic elements (MGEs) were enriched in the accessory genome versus the core genome. A systematic analysis for MGEs identified the first candidate *D. pigrum* prophage and insertion sequence. A systematic analysis for genetic elements that limit the spread of MGEs, including restriction modification (RM), CRISPR-Cas, and deity-named defense systems, revealed strain-level diversity in host defense systems that localized to specific genomic sites, including one RM system hot spot. Analysis of CRISPR spacers pointed to a wealth of MGEs against which *D. pigrum* defends itself. These results reveal a role for horizontal gene transfer and mobile genetic elements in strain diversification while highlighting that in *D. pigrum* this occurs within the context of a highly stable chromosomal organization protected by a variety of defense mechanisms.

**IMPORTANCE** Dolosigranulum pigrum is a candidate beneficial bacterium with potential for future therapeutic use. This is based on its positive associations with characteristics of health in multiple studies of human nasal microbiota across the span of human life. For example, high levels of *D. pigrum* nasal colonization in adults predicts the absence of Staphylococcus aureus nasal colonization. Also, *D. pigrum* nasal colonization in young children is associated with healthy control groups in studies of middle ear infections. Our analysis of 28 genomes revealed a remarkable stability of *D. pigrum* strains colonizing people in the United States across a 20-year span. We subsequently identified factors that can influence this stability, including genomic stability, phage predators, the role of MGEs in strain-level variation, and defenses against MGEs. Finally, these *D. pigrum* strains also lacked predicted virulence factors. Overall, these findings add additional support to the potential for *D. pigrum* as a therapeutic bacterium.

## INTRODUCTION

Evidence points to a prominent role for the benign nasal bacterium Dolosigranulum pigrum in structuring nasal microbiota beneficial to human health (1–30; reviewed in references 31, 32, 33, 34, 35, and 36). Individuals whose nasal microbiota is dominated by *D. pigrum* are less likely to be colonized by nasal pathobionts and are therefore at lower risk of invasive infections due to these microbes. For example, *D. pigrum* is inversely associated with Staphylococcus aureus in adult nostrils (5, 16, 28, 37). Also, the level of maternal *D. pigrum* is inversely associated with infant acquisition of S. aureus (38); in a small study, neonates who do not acquire S. aureus have a higher relative abundance of *D. pigrum* (39). During *in vitro* growth, *D. pigrum* inhibits S. aureus on agar medium, but not the reverse (28), suggesting *D. pigrum* might directly antagonize S. aureus *in vivo*. In addition, *D. pigrum* and nasal *Corynebacterium* species are frequently present in pediatric nasal microbiota when Streptococcus pneumoniae is absent (1, 8). Together, *D. pigrum* and Corynebacterium pseudodiphtheriticum robustly inhibit S. pneumoniae *in vitro* compared to either organism alone (28). As illustrated by these examples, nasal microbiota with higher levels of *D. pigrum*—usually alongside *Corynebacterium*—are often associated with health. Young infants with prolonged high levels of *D. pigrum* and *Corynebacterium* exhibit greater stability of their nasal microbiota and fewer respiratory tract infections (3, 4, 6, 11, 21). Also, higher levels of nasal *D. pigrum* and *Corynebacterium* are more common in healthy children than in children with pneumonia (12) or those with otitis media (1, 2, 15, 30).

In stark contrast to the steadily increasing data in support of *D. pigrum* as a candidate beneficial bacterium (40), there is a dearth of information about the basic biology of this Gram-positive organism, including the organization and stability of its genome. Ideally, bacterial strains with therapeutic potential display a reliably stable genome structure and have the capacity to resist horizontal gene transfer (HGT), since the latter might lead to unanticipated effects. The stability of bacterial genomes reflects a balance between competing factors, including invasion by mobile genetic elements (MGEs) and systems that defend against MGEs. MGEs play a key role in strain variation through acquisition and distribution of genes in the accessory genome. Analysis of the pangenome of multiple strains identifies core and soft-core gene clusters (GCs) common to all, or almost all, of the strains, respectively, and GCs present in smaller subsets of strains, which constitute the accessory genome (41, 42). Although accessory genes may result from gene loss, many are thought to be acquired via HGT. Counterbalancing this are key systems for defense against MGEs. These include well-described restriction modification systems, CRISPR-Cas systems, and the more recently identified, deity-named defense systems (43). Restriction modification (RM) systems distinguish intracellular DNA as self or nonself by virtue of specific methyl modifications within short linear sequences that allow for destruction of inappropriately methylated nonself DNA by endonuclease activity; the various RM systems are classified into types I, II, III, and IV. There are also other variations of DNA modification-based defense (44, 45). CRISPR-Cas systems mediate defense using a multistep process. Small fragments of foreign nucleic acids are first recognized as non-self and incorporated into the host genome between short DNA repeats, known as a CRISPR array. Subsequently, these fragments, now spacers within the array, are used as RNA guiding molecules for an endonuclease complex that recognizes and destroys DNA containing these sequences (46). The more recently identified deity-named defense systems consist of a set of 10 disparate antiphage/plasmid mechanisms that are often found clustered next to known defense genes (RM and CRISPR-Cas) (43) within defense islands (47) of bacterial genomes. Although deity-named defense systems have been shown to be active and limit phage/plasmid spread, their exact underlying modes of action remain to be deciphered. Collectively, these systems can protect bacteria from infection by phages and invasion by other MGEs, including plasmids and transposable elements, thus limiting the introduction of new genes and maintaining genomic stability.

Comparing genomic content and chromosomal organization of *D. pigrum* strains collected 20 years apart, and mostly in the United States, we identified the following characteristics: (i) highly similar strains circulating across 20 years; (ii) stable chromosomal synteny across the phylogeny; (iii) the first predicted *D. pigrum* prophage and insertion sequence; and (iv) a diverse collection of RM, deity-named defense and CRISPR-Cas systems incorporated at conserved chromosomal insertion sites across strains. Together, these reveal a stable synteny and a high-level of sequence conservation within the *D. pigrum* core genome, along with an open pangenome and active defense against HGT.

## RESULTS

### Detection of highly similar *Dolosigranulum pigrum* strains over a 20-year span.

To identify genomic shifts in *D. pigrum* strains currently circulating in human nasal microbiota compared to strains from approximately 20 years ago, we collected 17 new nostril isolates of *D. pigrum* from volunteers in 2017 and 2018 and sequenced the genomes of these isolates using SMRTSeq (PacBio), fully circularizing 14 (Table 1). We compared these 17 new genomes to 11 described genomes (28), 9 of which are from strains collected in the late 1990s (48). This refined existing and uncovered new information about the basic genomic characteristics of *D. pigrum* (see Table S1 in the supplemental material).

**TABLE 1:** Source information for the 28 *D. pigrum* strains and quality description for the 17 newly SMRT-sequenced closed genomes^*a*^

Original strain name	Internal reference	Yr isolated	Human body site	Location	Age (yrs)	NCBI assembly ID	Source or reference	Realigned bases (%)^*b*^	Coverage (fold)
ATCC 51524	NA	1988	Spinal cord	UK	?	GCF_000245815.1	135		
KPL1914	KPL1914	2010	Nostril	MA	Adult	GCA_003263915.2	28		
CDC39-95	KPL1922	1995	NP	CN	3	GCF_003264145.1	48		
CDC2949-98	KPL1930	1998	NP	AZ	?	GCF_003264135.1	48		
CDC4294-98	KPL1931	1998	Blood	SC	<1	GCF_003264085.1	48		
CDC4420-98	KPL1932	1998	Blood	TN	11	GCF_003264065.1	48		
CDC4545-98	KPL1933	1998	NP	AZ	?	GCF_003264045.1	48		
CDC4709-98	KPL1934	1998	Eye	GA	<1	GCA_003264015.2	48		
CDC4199-99	KPL1937	1999	Blood	GA	∼2	GCF_003264005.1	48		
CDC4791-99	KPL1938	1999	NP	AZ	?	GCF_003263975.1	48		
CDC4792-99	KPL1939	1999	NP	AZ	?	GCF_003263965.1	48		
KPL3033	KPL3033	2018	Nostril	MA	18–30	GCA_017655925.1	This study	92.61*	498
KPL3043	KPL3043	2018	Nostril	MA	7–12	GCA_017655905.1	This study	92.40*	582
KPL3050	KPL3050	2018	Nostril	MA	31–60	GCA_017655885.1	This study	92.11*	475
KPL3052	KPL3052	2018	Nostril	MA	3–6	GCA_017655865.1	This study	92.15*	382
KPL3065	KPL3065	2018	Nostril	MA	7–12	GCA_017655845.1	This study	91.73*	460
KPL3069	KPL3069	2018	Nostril	MA	7–12	GCA_017655825.1	This study	88.13*	372
KPL3070	KPL3070	2018	Nostril	MA	31–60	GCA_017655785.1	This study	91.85*	271
KPL3077	KPL3077	2018	Nostril	MA	7–12	GCA_017655765.1	This study	91.60	351
KPL3084	KPL3084	2018	Nostril	MA	31–60	GCA_017655745.1	This study	90.24*	433
KPL3086	KPL3086	2018	Nostril	MA	<3	GCA_017655725.1	This study	91.30*	342
KPL3090	KPL3090	2018	Nostril	MA	7–12	GCA_017655685.1	This study	90.72*	423
KPL3246	KPL3246	2018	Nostril	MA	7–12	GCA_017655805.1	This study	92.47*	578
KPL3250	KPL3250	2018	Nostril	MA	7–12	GCA_017655665.1	This study	92.63*	501
KPL3256	KPL3256	2018	Nostril	MA	7–12	GCA_017655645.1	This study	92.84	530
KPL3264	KPL3264	2018	Nostril	MA	7–12	GCA_017655705.1	This study	87.61	342
KPL3274	KPL3274	2018	Nostril	MA	7–12	GCA_017655945.1	This study	87.41*	574
KPL3911	KPL3911	2017	Nostril	MA	<3	GCA_017655965.1	This study	87.13*	595

a NA, not applicable; NP, nasopharynx.

b Percent realigned bases (from Realignment to Draft Assembly). *, circularized genome.

10.1128/mSystems.00425-21.1 TABLE S1 Pangenome contribution of all 28 *D. pigrum* genomes. Download Table S1, PDF file, 0.04 MB. Copyright © 2021 Flores Ramos et al. 2021 Flores Ramos et al. https://creativecommons.org/licenses/by/4.0/ This content is distributed under the terms of the Creative Commons Attribution 4.0 International license.

**FIG 1:**
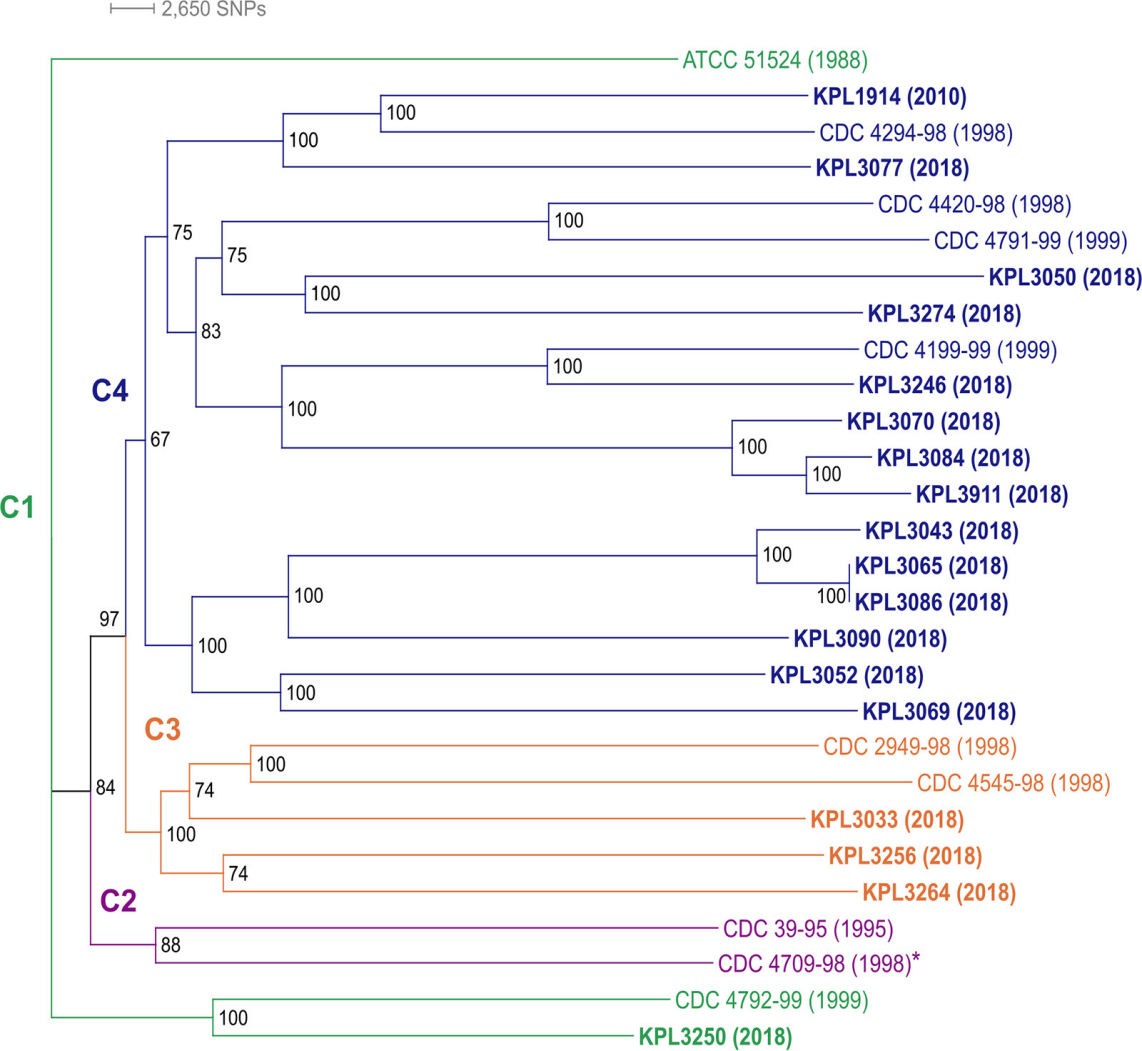
Dolosigranulum pigrum strains collected 20 years apart are phylogenetically similar. This maximum-likelihood core-gene-based phylogeny shows recently collected strains (bold), mostly from 2018, and strains collected before 2000 intermingled in three of the four distinct clades (clades C1 to C4 are color coded, and the year of collection is in parentheses). Strains separated by 18 to 19 years grouped together in terminal clades: KPL1914 and CDC4294-98, KPL3246 and CDC4199-99, and KPL3250 and CDC4792-99. The genomes of strains in boldface plus strain CDC4709-98 (asterisk) are closed. Strains KPL3065 and KPL3086 were collected from two different individuals and have almost identical genomes, differing by just 4 core SNPs and 6 gene clusters (4 and 2 in KPL3086 and KPL3065, respectively). We created this unrooted phylogeny using the concatenated alignment of 1102 conservative single-copy core GCs (see Fig. S3A), a GTR+F+R3 substitution model of evolution, 553 maximum-likelihood searches, and 1,000 ultrafast bootstraps with IQ-Tree v.1.

To assess the similarity of these 28 *D. pigrum* strains, we generated a phylogenomic tree based on 1,102 single-copy core GCs (Fig. 1). Some of the terminal clades include strains collected during different decades. The average number of pairwise single nucleotide polymorphisms (SNPs) among isolates collected approximately 20 years apart was similar to that among isolates collected recently (21,754 versus 20,834) (see Table S2A). Thus, closely related strains of *D. pigrum* have circulated among people in the United States over a span of time that has an upper bound of 20 years and a lower bound of 8 to 13 years. (This lower bound allows for the possibility that the recent isolates were stably acquired in infancy since most of the 2018 strains were from children in the 7- to 12-year age range.) Alloiococcus otitis (49) is the closest genome-sequenced bacterium to *D. pigrum* in 16S rRNA gene phylogenies. *A. otitis* ATCC 51267 shared 789 core GCs with the *D. pigrum* strains (see Fig. S1A). Using these 789 core GCs, we constructed a phylogenomic tree with *A. otitis* as an outgroup (see Fig. S1B and C). In contrast to the *D. pigrum*-only phylogeny (Fig. 1), the phylogeny including *A. otitis* displayed poor support for many of the branches within the *D. pigrum* clade. This is likely due to the reduced number of SNPs among *D. pigrum* strains when using only the 789 GCs shared with *A. otitis* (see Table S2A). Therefore, we based subsequent inferences on the *D. pigrum*-only phylogeny.

10.1128/mSystems.00425-21.2 TABLE S2 Pairwise SNP analysis and MASH analysis. Download Table S2, XLSX file, 0.06 MB. Copyright © 2021 Flores Ramos et al. 2021 Flores Ramos et al. https://creativecommons.org/licenses/by/4.0/ This content is distributed under the terms of the Creative Commons Attribution 4.0 International license.

10.1128/mSystems.00425-21.6 FIG S1 A core-gene phylogenomic tree including Alloiococcus otitis ATCC 51267 as an outgroup has poor resolution. (A) The 789 GCs shared between the BDBH, COG triangle, and OMCL algorithms were used to determine the single-copy GCs shared between the 28 *D. pigrum* genomes and the *A. otitis* outgroup using GET_HOMOLOGUES v. 3.1.4. (B) The phylogenomic tree with ancestral distance displayed. (C) The phylogenomic tree as a cladogram. *A. otitis* is the most closely related genome-sequenced taxon to *D. pigrum* based on 16S rRNA gene sequence in both the eHOMD and the Living Tree Project (P. Yarza, M. Richter, J. Peplies, J. Euzeby, R. Amann, K. H. Schleifer, W. Ludwig, F. O. Glöckner, R. Rosselló-Móra. Syst Appl Microbiol. 31:241–250, 2008, [doi: 10.1016/j.syapm.2008.07.001]; P. Yilmaz, L.W. Parfrey, P. Yarza, J. Gerken, E. Pruesse, C. Quast, T. Schweer, J. Peplies, W. Ludwig, F. O. Glöckner. Nucleic Acids Res. 42:D643–D648, 2014 [doi: 10.1093/nar/gkt1209]). We generated a phylogenomic tree based on 789 concatenated core GCs shared among *A. otitis* ATCC 51267 (NZ_JH992957) and the 28 *D. pigrum* genomes with IQ-Tree using a GTR+F+R6 substitution model (BIC value 5743936.9087), 300 ML searches (ML = −2868528.1268) and 1,000 ultrafast rapid Bootstraps. There is a deep branch separating *A. otitis* from all the *D. pigrum* strains. However, many of the ancestral branches within the *D. pigrum* clade are poorly supported. Repeated construction resulted in phylogenies with poorly supported branching likely due to poor SNP resolution (see Table S1A), suggesting the need for a better outgroup for *D. pigrum*. Download FIG S1, PDF file, 0.2 MB. Copyright © 2021 Flores Ramos et al. 2021 Flores Ramos et al. https://creativecommons.org/licenses/by/4.0/ This content is distributed under the terms of the Creative Commons Attribution 4.0 International license.

10.1128/mSystems.00425-21.8 FIG S3 The conservative core genome of *D. pigrum* has 1102 GCs with a very high degree of nucleotide conservation among strains and the accessory genome size varies but the functions are similar among the 28 genomes. (A) We used the intersection of the BDBH, COG triangle, and OMCL algorithm results to determine a conservative core genome of 1102 single-copy GCs using the 28 *D. pigrum* genomes on GET_HOMOLOGUES v. 3.1.4. (B) We determined the GCs present in the core (*n* = 28), soft core (28 > *n* ≥ 26), shell (26 > *n* ≥ 3), and cloud (*n* ≤ 2) using the results from the OMCL and COG triangle algorithms. Of the 3700 total GCs, 30.6% are core (1,134/3,700), 5.41% are soft core (200/3,700), 22.8% are shell (845/3700), and 41.1% are cloud (1,521/3,700). (C) All paired strains share above 97.58% average nucleotide identity. We based this on the average nucleotide identity of the homologues determined using the OMCL algorithm. (D) The *D. pigrum* Anvi’o pangenome is similar to the GET_HOMOLOGUES pangenome. The biggest difference between the GET_HOMOLOGUES- and Anvi’o-defined pangenome was the migration of many of the GET_HOMOLOGUES cloud GCs to the Anvi’o multicopy core, causing the pangenome size to drop from 3,700 to 2,905 GCs. Occupancy of the GCs among the 28 genomes is displayed with strains collected in 2018 and 2017 (blue), strains from the CDC (pink), the ATCC strain (gold), and the strain collected in 2010 (green). Strains are arranged based on the phylogeny from Fig. 1 (right). With Anvi’o, 44.7% of the GCs (1,298/2,905) were in the core, 3.1% (90/2,905) in the soft core (green), 27.7% (805/2,905) in the shell (blue), and 24.5% (712/2,905) in the cloud (purple). A closer inspection of the core also showed 38.2% (1111/2,905) of the GCs are in the conservative single-copy (SC) core (red) and 6.4% (187/2,905) are in the multicopy (MC) core (dark red). The interactive Anvi’o pangenome can also be found on our GitHub (https://github.com/KLemonLab/DpiMGE_Manuscript/blob/master/SupplementalMethods_Anvio.md). (E) The accessory genome size varies but the functions are similar among the 28 *D. pigrum* genomes. Using the functional annotations of the accessory GCs identified by Anvi’o (defined as “shell” plus “cloud” bins), we found that most of the accessory GCs among all of our genomes either had no annotation (“unclassified,” 53.6%), had an ambiguous COG categorization (“ambiguous”, 6.6%) or belonged to the uninformative S or R COG category (“uninformative,” 2.6%). Only 37.2% of the GCs in the accessory genome had informative COG annotations (colored categories). (F) Of the informative GCs, there was a similar proportional distribution of accessory functions among all 28 genomes with a large fraction dedicated to “defense mechanisms” (olive) and “carbohydrate transport and metabolism” (khaki). The size of the accessory genomes varied among the strains with CDC4294-98 and KPL3090 having the largest accessory size compared to KPL3065 and KPL3086 with the smallest. Horizontal bars indicate clades: clade 1, green; clade 2, purple; clade 3, orange; and clade 4, blue. Download FIG S3, PDF file, 1.1 MB. Copyright © 2021 Flores Ramos et al. 2021 Flores Ramos et al. https://creativecommons.org/licenses/by/4.0/ This content is distributed under the terms of the Creative Commons Attribution 4.0 International license.

### The chromosome of *D. pigrum* exhibits conserved synteny across a phylogeny spanning 20 years.

**FIG 2:**
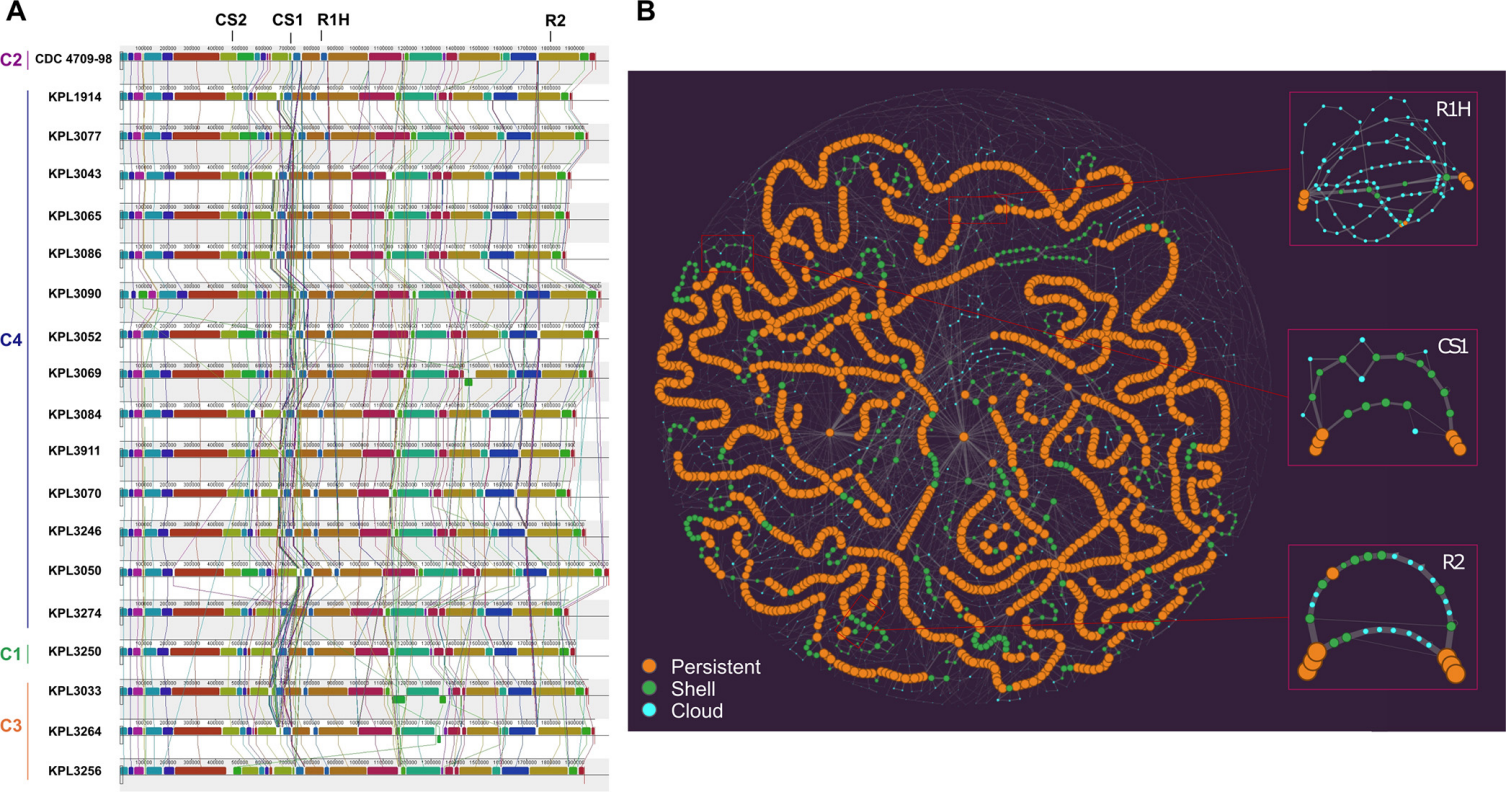
*D. pigrum* displays conserved chromosomal synteny. (A) A MAUVE alignment of 19 closed *D. pigrum* genomes, with representatives from the four major clades in Fig. 1, shows a conserved order of chromosomal blocks across the phylogeny of strains collected 20 years apart. Vertical bars represent clades: clade 1, green; clade 2, purple; clade 3, orange; and clade 4, blue. CS1 and CS2 designate the CRISPR-Cas sites (Fig. 8), R1H represents the RM system insertional hot spot and R2 represents the site containing either a type II m5C RM system or a type IV restriction system (Fig. 7). (B) This PPanGGOLiN partitioned pangenome graph displays the overall genomic diversity of the 28 *D. pigrum* genomes. Each graph node corresponds to a GC; the node size is proportional to the total number of genes in a given cluster, and the node color represents the PPanGGOLiN assignment of GCs to the partitions: persistent (orange), shell (green), and cloud (blue). Edges connect nodes that are adjacent in the genomic context and their thickness is proportional to the number of genomes sharing that neighboring connection. The insets on the right depict subgraphs for sites R1H, CS1, and R2 showing several branches corresponding to multiple alternative shell and cloud paths. These sites with higher genomic diversity are surrounded by longer regions with conserved synteny, i.e., long stretches of consecutive persistent nodes (GCs). The static image depicted here was created with the Gephi software (https://gephi.org) (133) using the ForceAtlas2 algorithm (134) with the following parameters: scaling = 20,000, stronger gravity = true, gravity = 6.0, LinLog mode = true, and edge weight influence = 2.0.

Based on the observed similarity of circulating strains over time, we hypothesized there would be a high-level of genomic stability across the *D. pigrum* phylogeny. To test this, we compared chromosomal synteny across the four major clades in the *D. pigrum* phylogeny using 19 strains with closed genome sequences (highlighted in bold or with an asterisk [*] in Fig. 1), including representative strains collected in 1998, 2010, 2017, and 2018. A MAUVE alignment (50, 51) of these 19 genomes starting at the *dnaA* gene revealed a remarkable conservation of the overall chromosomal structure with no visible shifts in the position of large blocks of sequence (Fig. 2A). Dispersed among these blocks are regions with higher numbers of insertions and deletions (indels) (Fig. 2A; see also Fig. S2A).

10.1128/mSystems.00425-21.7 FIG S2 A 100-kb region with a higher frequency of indels and a second region with a higher frequency of phage and plasmid integration events. (A) Closer inspection of positions ∼650000 to ∼750000 in the MAUVE alignment showed the presence of three sites containing sequencing heterogeneity (HS for heterogeneity sites) bounded by >10 kb blocks of homology. The first site (HS1), between *abgT* (p-aminobenzoyl-glutamate transport protein) and *kynB* (kynurenine formamidase), contained a PTS (mannitol) system for metabolism. However, the extent of completeness of this PTS system varied among the *D. pigrum* isolates and was absent in KPL3274 and KPL3256. The next site (a few kb downstream of HS1), between *yxdM* (ABC transporter permease protein YxdM) and *ghrA* (glyoxylate/hydroxypyruvate reductase A), contained one of the two possible CRISPR-Cas systems, with all genomes having at least one type (CS1; Fig. 8A and B). Finally, the third site (HS2), between *nth* (endonuclease III) and *clsA_1* (cardiolipin synthase A), variably harbored two types of aspartate and threonine synthase genes. One type in KPL3033 and KPL3052; none in KPL1914, KPL3077, and KPL3274; and a variation of the second type among the remaining genomes. Also indicated on this MAUVE alignment are the second of the two CRISPR-Cas insertion sites (CS2) and the location of the pUB110 insertion, which is in a region with a higher frequency of phage and plasmid integration events. Vertical bars represent clades: clade 1, green; clade 2, purple; clade 3, orange; and clade 4, blue. (B) No autonomous plasmids were detected among the 28 genomes using the Gram-positive plasmid database, PlasmidFinder. However, as illustrated with this map from *D. pigrum* KPL3043, we detected a sequence match to pUB110 (GenBank ID NC_001384.1), a plasmid originally isolated from S. aureus, integrated in four of the *D. pigrum* isolates (KPL3043, KPL3065/KPL3086 in clade 4 and CDC4709-98 in clade 2; Fig. 1). This included a perfect match (708/708 bp) to the *repB* gene (blue) of pUB110, which was located adjacent to three other genes (cyan) that are also present in pUB110 and predicted to encode a kanamycin nucleotidyltransferase ANT(4′)-lb (*knt*), a bleomycin resistance protein (*ble*), and a plasmid recombination protein (*pre-2*). Inspection of the region adjacent to the genes from pUB110, at position ∼1.45 Mb in the MAUVE alignment (A), showed evidence of plasmid elements present in 11 of the other closed *D. pigrum* genomes, suggesting this region has a higher frequency of phage and plasmid integration events. For example, a 73% (288/391 bp) match to a different (non-pUB110) *repB* gene present in plasmids of other closely related *Firmicutes* including Lactobacillus plantarum (KU707950) (X. Ma, J. Li, Y. Xiong, Z. Zhai, F. Ren, Y. Hao. Curr Microbiol. 73:820–826, 2016 [doi: 10.1007/s00284-016-1124-7]) and Lactobacillus curvatus (CP031007) (R.M. Prechtl, https://www.researchgate.net/publication/337103133_Formation_and_structure_of_exopolysaccharides_of_meat_starter_cultures). A subset of the genomes, including six from clade 4 (KPL3077, KPL3090, KPL3069, KPL3084, KPL3911, and KPL3070) and two from clade 3 (KPL3264 and KPL3256), had an *ltrA* gene (group II reverse transcriptase/maturase) at this location. Download FIG S2, PDF file, 1.0 MB. Copyright © 2021 Flores Ramos et al. 2021 Flores Ramos et al. https://creativecommons.org/licenses/by/4.0/ This content is distributed under the terms of the Creative Commons Attribution 4.0 International license.

### *D. pigrum* has a core genome that has leveled off, an open pangenome, and a high degree of conservation at the amino acid and nucleotide level.

**FIG 3:**
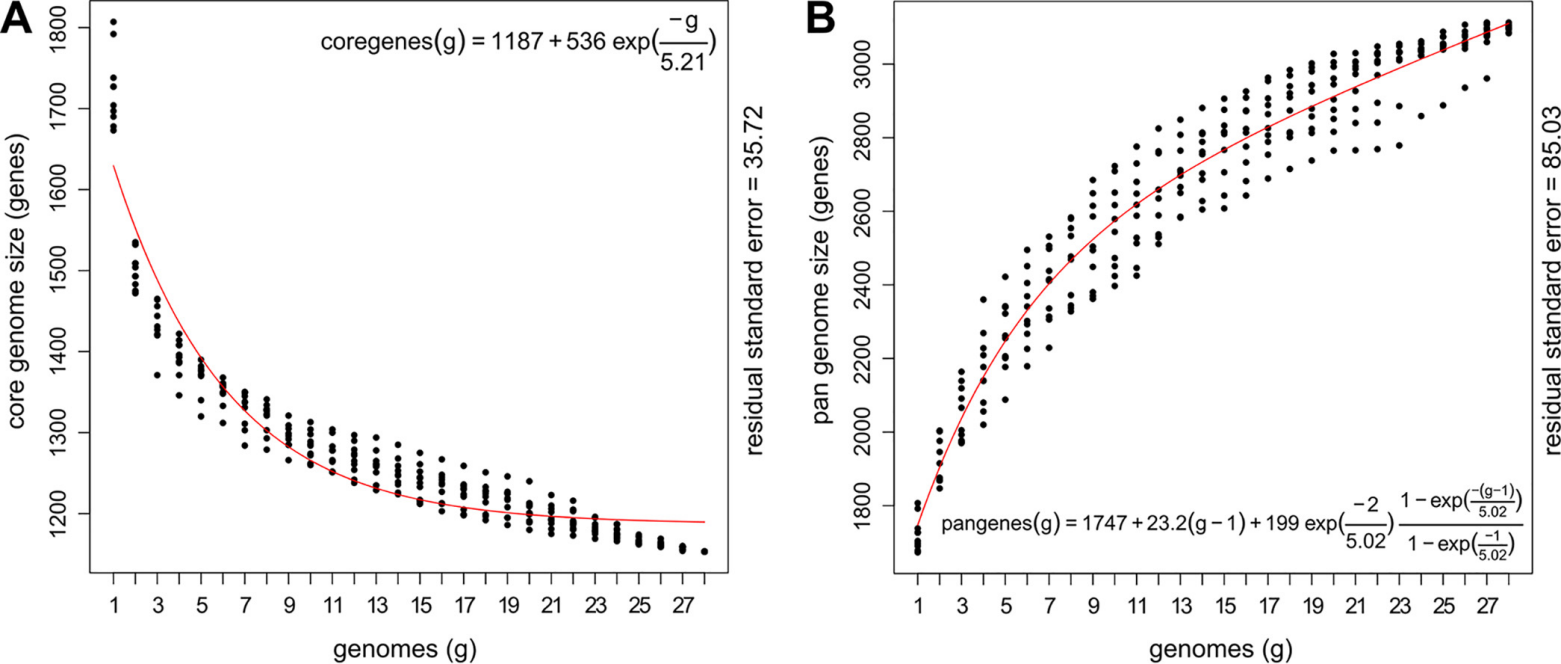
The *D. pigrum* core genome levels off, and the pangenome remains open. (A and B) The *D. pigrum* core (*n* = 28) genome started to level off after 17 genomes, as predicted using a Tettelin curve fit model (red line) (A), whereas, with 28 genomes, the pangenome continued to increase in gene clusters with each additional genome (B). *D. pigrum* core (A) and pangenome (B) size estimations were based on 10 random genome samplings (represented by black dots) using the OMCL algorithm defined gene clusters in GET_HOMOLOGUES v.3.1.4.

Analysis of all 28 *D. pigrum* genomes revealed a conservative core of 1,102 single-copy GCs, as defined by the intersection of results from three algorithms, including bidirectional best hits (BDBH) (see Fig. S3A). A core of 1,134 GCs was defined by the intersection of two algorithms when BDBH was excluded (see Table S1 and Fig. S3B). The *D. pigrum* core genome has leveled off in size (Fig. 3A). Meanwhile, the pangenome continued to increase, with each additional genome (Fig. 3B) reaching 3,700 GCs (see Fig. S3B); of these, 30.6% (1,134/3,700) are core. The average number of coding sequences (CDS) per genome was 1,765 and, on average, the core constituted ∼64% (1,134/1,765) of the CDS in each individual genome (see Table S1). These results from GET_HOMOLOGUES (42) generally agreed with those from Anvi’o (52, 53), allowing us to leverage Anvi’o for additional analyses. In the Anvi’o-derived single-copy core (38.2%; Fig. S3D), 89.4% (993/1,111) of the GCs had a functional homogeneity index score ≥0.98, indicating a high degree of conservation at the amino acid level. This fits with an average nucleotide identity (ANI) over 97.58% for all 28 genomes (see Fig. S3C), matching earlier findings with 11 strain genomes (28). Moreover, two sets of three recently collected strains each shared over 99% ANI, as well as similar accessory Clusters of Orthologous Group (COG) annotations (see Fig. S3E). This revealed highly similar strains in the nasal microbiota of different individuals in Massachusetts. Of these, two strains collected from different people were nearly identical, differing by just 4 core SNPs and 6 GCs (4 and 2 in KPL3086 and KPL3065, respectively) with a MASH-distance of 3.10E-05 (*P = *0; Table S2B). (Henceforth, we refer to these two strains as KPL3065/KPL3086.) In contrast KPL3086 and KPL3043, which are in that same distal clade in Fig. 1, have a MASH distance of 0.0045 (*P = *0).

### The *D. pigrum* accessory genome is enriched for gene clusters involved in mobilome and host defense.

**FIG 4:**
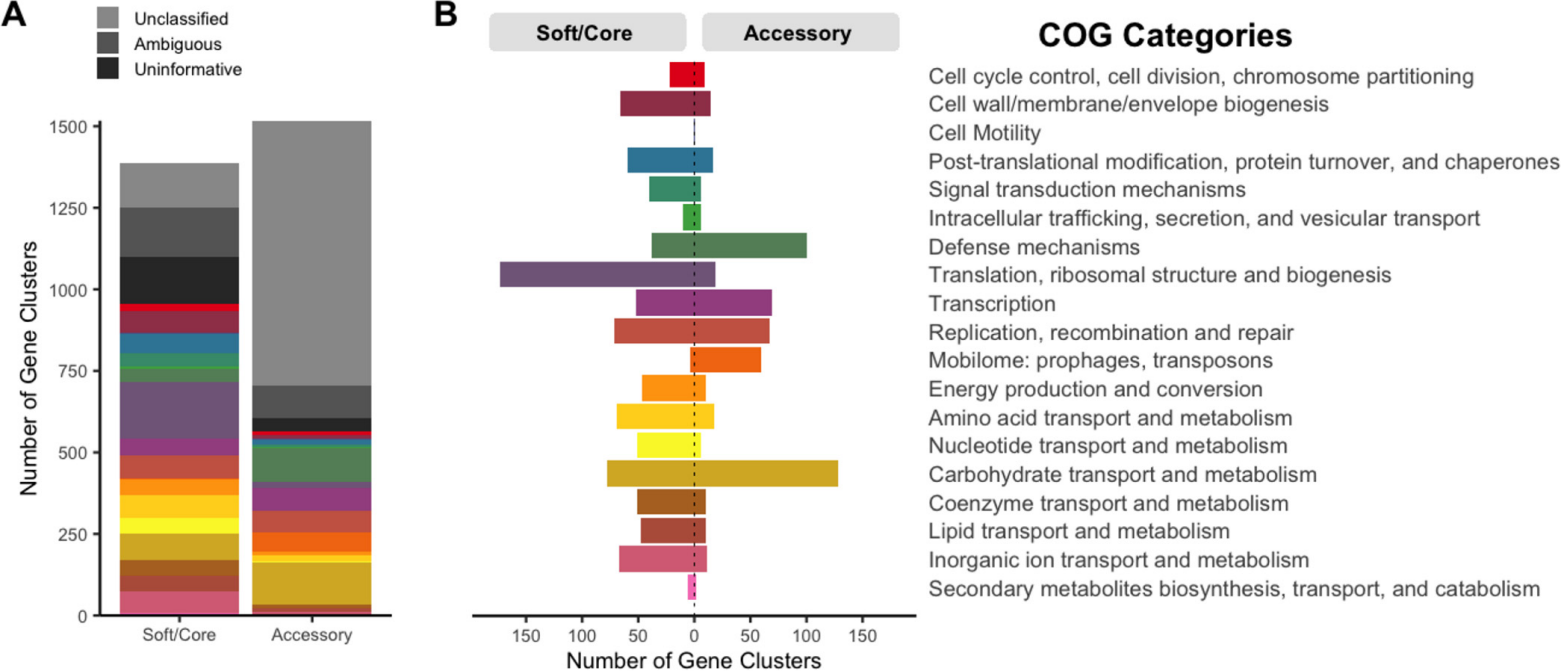
The accessory genome of *D. pigrum* has functional enrichment for defense mechanisms, mobilome, and carbohydrate transport and metabolism genes. (A) Of the total 49,412 individual genes identified across the 28 analyzed genomes, up to 8,242 genes (16.7%) lacked a COG annotation, 5,221 (10.6%) had an ambiguous COG category annotation (more than one COG category), and 4,448 (9.0%) had an uninformative annotation (belonging to the S or R COG category). At the gene cluster (GC) level, only 37.2% of the 1,517 GCs present in the accessory genome had an informative COG assignment compared to 68.7% of the 1,388 GCs in the soft/core. (B) The number of GCs present in the accessory genome was severalfold higher than in the soft/core for the following informative COG assignments (colored categories): defense mechanisms (olive, 2.60-fold), mobilome: prophages, transposons (orange, 14.88-fold), and carbohydrate transport and metabolism (khaki, 1.66-fold). This was determined using the COG functional annotations defined in our Anvi’o analysis of the soft/core (“core” and “soft core” bins) versus accessory (“shell” and “cloud” bins). Since many GCs have individual genes with distinct COG annotations each individual gene was counted as 1/*x*, with *x* being the number of genes in each GC.

Of the 49,412 individual genes identified across the 28 genomes, 63.8% (31,501/49,412) had informative calls to a single functional COG annotation (i.e., their assignment corresponds to a single COG category other than S or R) (54, 55) (Fig. 4A). Using Anvi’o, we observed that GCs involved in mobilome, in defense mechanisms, and in carbohydrate transport and metabolism were overrepresented in the accessory compared to the core genome (Fig. 4B). GCs classified to these three COG categories accounted for 3.9, 6.6, and 8.5% of the *D. pigrum* accessory genome, respectively. The proportion of accessory functions was similar among all strains, but the sizes of their accessory genomes varied (see Fig. S3E and F). Because genome stability is relevant to suitability of a candidate beneficial microbe for therapeutic use, we focused subsequent analysis on the predicted mobilome and defense mechanisms.

### *D. pigrum* hosts distinct integrated phage elements, insertional elements, and a group II intron.

**FIG 5:**
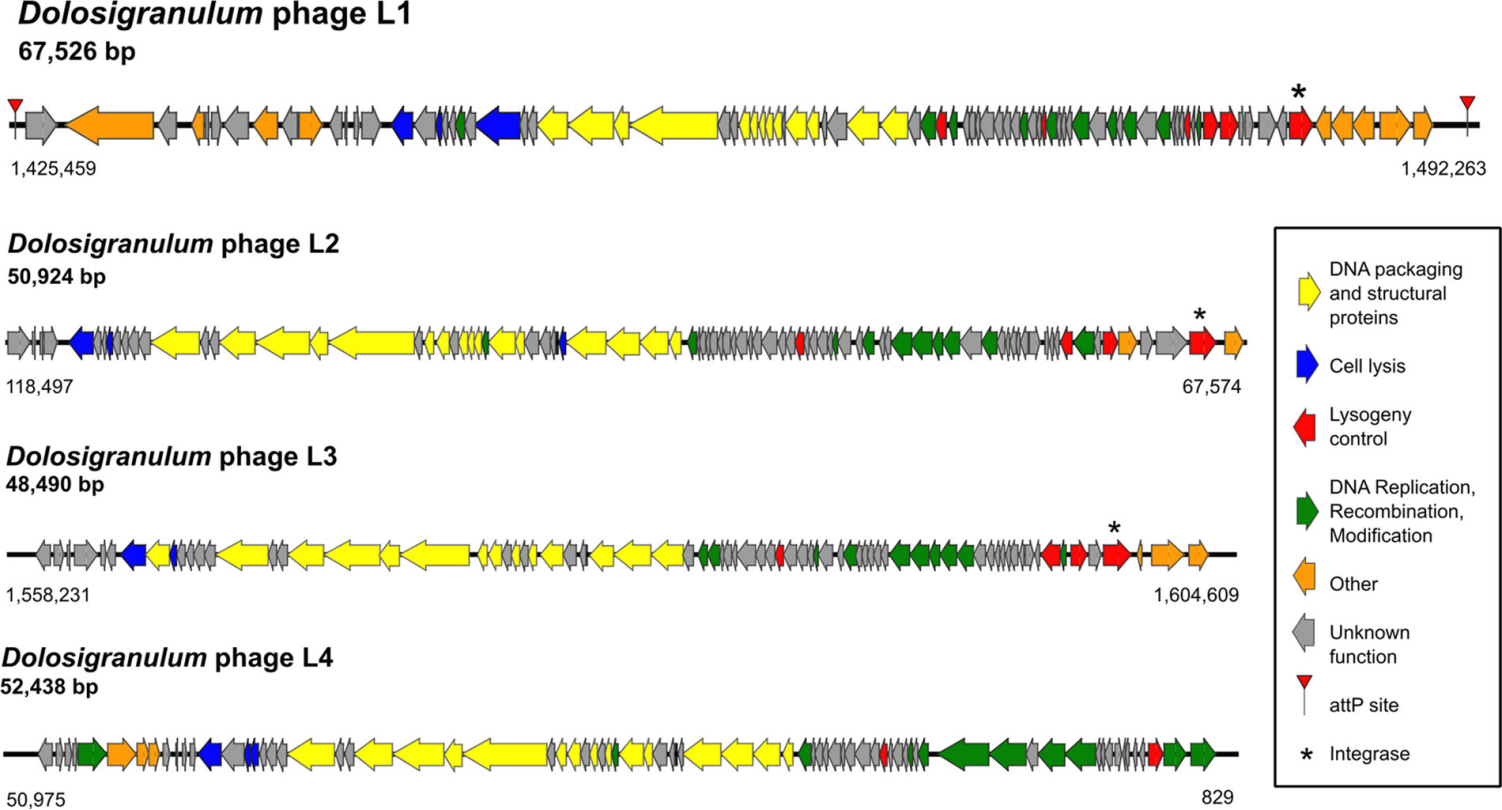
*D. pigrum* has an intact prophage. Map of the four predicted prophages: *Dolosigranulum* phage L1 from KPL3069, L4 from KPL3256, and L2 and L3 from KPL3090. The most complete prophage was L1 from KPL3069 with an intact integrase and two *attP* sites. All of the putative phages exhibited a typical life cycle-specific organization, with lytic genes on one side and lysogenic genes on the other. We detected phage elements using the PHASTER database on 8 November 2018.

Of the total GCs in the pangenome, 2.2% were predicted to be part of the mobilome. MGEs can negatively affect genome stability and can positively affect strain diversification. Therefore, we systematically searched for various types of MGEs, including phage elements, plasmids, and insertional elements that interact with *D. pigrum.* First, using the Phage Tool Enhanced Release (PHASTER) database (56, 57), we identified four distinct, and mostly intact, integrated phage elements, i.e., prophages (Fig. 5). We gave these the provisional names *Dolosigranulum* phage L1 through L4. All four were in the size range common for *Firmicutes* phages and had a life cycle-specific organization of its CDS with lytic and lysogenic genes separated (Fig. 5) (58–60). Predicted prophage L1 from *D. pigrum* KPL3069 was the most intact with two attachment (*attP*) sites and an intact integrase most similar to that of the Streptococcus prophage 315.2 (NC_004585; E value 7.85e-69) (61). Prophages L2 and L3 from *D. pigrum* KPL3090 also had intact integrases, with similarity to other streptococcal phages, but lacked distinguishable *attP* sites. Beyond these similarities, other CDS from L1 to L4 displayed few and dissimilar matches to known phage elements (see Text S1), indicating that *D. pigrum* hosts a distinct set of lysogenic phage that are expected to have a limited host range.

10.1128/mSystems.00425-21.10 TEXT S1 Supplemental analysis of genetic elements and defense systems. Download Text S1, PDF file, 0.10 MB. Copyright © 2021 Flores Ramos et al. 2021 Flores Ramos et al. https://creativecommons.org/licenses/by/4.0/ This content is distributed under the terms of the Creative Commons Attribution 4.0 International license.

Second, using the Gram-positive plasmid database PlasmidFinder (62), we detected no autonomous plasmids. However, a nearly complete fragment of the S. aureus plasmid pUB110 is integrated in the chromosome of four strains and includes a gene encoding kanamycin resistance (see Fig. S2B). This prompted a systematic search for antibiotic resistance genes using the Comprehensive Antibiotic Resistance Database in the Resistance Gene Identifier (CARD-RGI) (63, 64). Of the 28 genomes, 6 are predicted to encode antibiotic resistance genes for erythromycin and/or kanamycin, which are located within a CRISPR array or the integrated plasmid, respectively (see Text S1).

**FIG 6:**
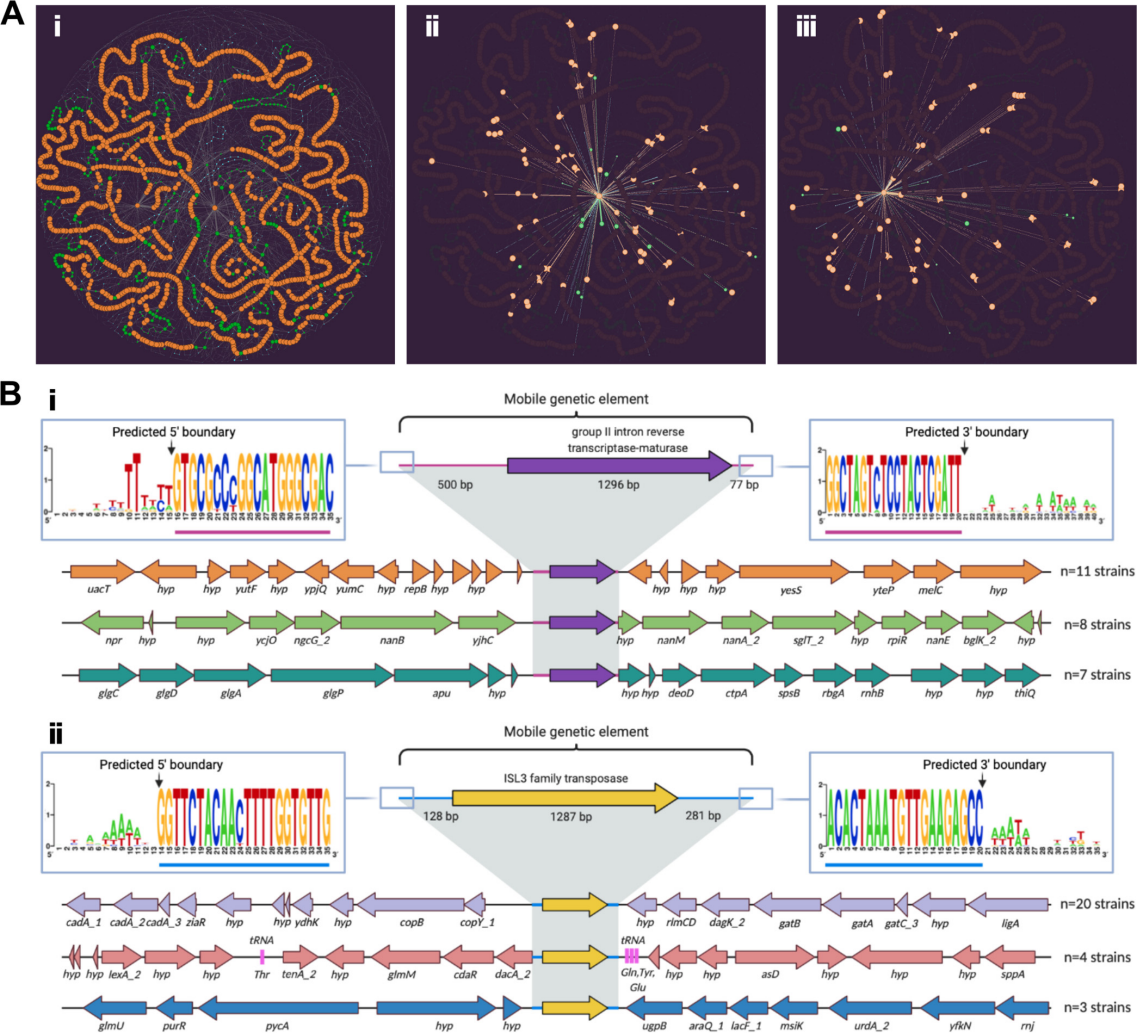
*D. pigrum* genomes host a few highly prevalent MGEs. (Ai) On the PPanGGOLiN partitioned pangenome graph for the 28 *D. pigrum* genomes, we highlight the neighboring connections for the persistent GC of a predicted group II intron reverse transcriptase-maturase (Aii; purple in panel Bi) and a predicted ISL3 family transposase (Aiii; yellow in panel Bii). Each graph node corresponds to a GC; node size is proportional to the total number of genes in a given cluster; and node color represents PPanGGOLiN assignment of GCs to the partitions: persistent (orange), shell (green), and cloud (blue). Edges connect nodes that are adjacent in the genomic context and their thickness is proportional to the number of genomes sharing that neighboring connection. In panels Aii and Aiii, only the adjacent neighboring nodes and edges for each of the depicted GCs are contrast colored against the background pangenome graph. (B) Most common genomic neighborhoods for the predicted group II intron reverse transcriptase-maturase (Bi) and the ISL3 family transposase (Bii). OCTAPUS (https://github.com/FredHutch/octapus) identified the chromosomal coordinates of each MGE integration event in individual strains, and groupings of colocated genes residing within the same neighborhood structure across strains were visualized using Clinker (https://github.com/gamcil/clinker). ClustalOmega alignments of flanking regions across groupings revealed predicted terminal sequence boundaries (consistent 5′–3′ sequences across integration events) for each MGE. The three most common genomic loci for each MGE were rendered using BioRender.

Third, we identified GCs predicted to be either transposases (eight) or integrases (five) using a multistep approach (see Table S3). Transposases are thought to function both as detrimental, selfish genetic elements that can disrupt important genes and as diversifying agents that can provide benefit to host cells through gene activation or rearrangements (65, 66). Among the 26 genomes containing at least one transposase CDS, the mean was 4.42 (median, 3.5), with a maximum of 13 per genome. Transposases were more prevalent and abundant than integrases (see Table S3). One of the predicted transposases was the GC containing the third largest number of sequences. This is consistent with reports that genes encoding transposases are the most prevalent protein-encoding genes detected across the tree of life when accounting for both ubiquity and abundance (67). We detected 74 intact instances of this most common transposase, an ISL3 family transposase with similarity to ISSau8, across 22 of the *D. pigrum* genomes with a mean (median) of 3.36 (2) and a maximum of 11 copies per genome (GC_00000003; Table S3). As shown on the PPanGGOLin graph (Fig. 6Ai), this transposase is inserted at multiple different sites within and across the genomes (Fig. 6Aiii; see also Table S3). The most common of these is likely the ancestral insertion site (Fig. 6Bii). The absence of a cotraveling CDS is consistent with this ISL3 family transposase being part of an insertional sequence (IS). According to standards for IS nomenclature, we propose the name ISDpi1 (66).

10.1128/mSystems.00425-21.3 TABLE S3 Predicted *D. pigrum* transposases, integrases and group II intron. Download Table S3, PDF file, 0.2 MB. Copyright © 2021 Flores Ramos et al. 2021 Flores Ramos et al. https://creativecommons.org/licenses/by/4.0/ This content is distributed under the terms of the Creative Commons Attribution 4.0 International license.

Fourth, the PPanGGOLin graph (68) revealed insertion of a predicted group II intron reverse transcriptase-maturase at multiple sites across multiple *D. pigrum* genomes (Fig. 6Aii and Bi; see also Table S3). Group II introns are MGEs commonly found in bacterial genomes that consist of a catalytic RNA and an intron-encoded protein that assists in splicing and mobility (69). Like transposases, group II introns can play both detrimental and beneficial roles within their host. We detected this intron-encoding GC in all 28 genomes with a mean (median) of 4.7 (3.5) and range of 1 to 14 copies per genome. This GC contained the highest number of individual gene sequences of any GC with 132 (GC_00000001; Table S3). It is most closely related to the bacterial class C intron-encoded protein from La.re.I1 in Lactobacillus reuteri with 44% identity and 65% similarity over 419 amino acids (70). These data are consistent with an intact bacterial reverse transcriptase/maturase expected to facilitate splicing and mobility of the group II intron (69).

### A systematic search identifies multiples types of defense systems to protect *D. pigrum* from MGEs.

The enrichment for defense mechanisms in the accessory genome of *D. pigrum* is combined with the relative paucity of plasmids and prophages among *D. pigrum* genomes. Based on this, we performed a systematic search of the pangenome for known bacterial host defense systems, including RM, deity-named defense, and CRISPR-Cas systems.

### *D. pigrum* harbors a diverse collection of RM systems.

**FIG 7:**
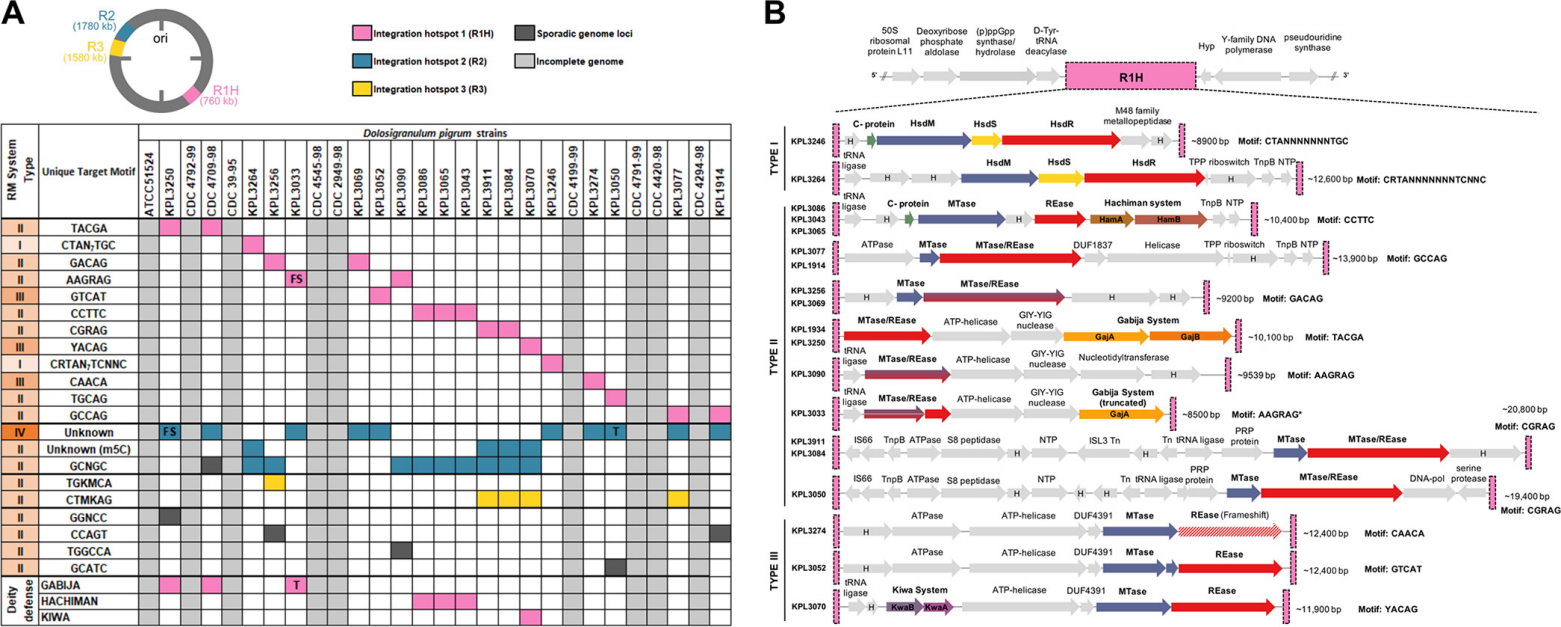
*D. pigrum* hosts a diverse collection of restriction-modification (RM) systems at three distinct loci. (A) Conserved methyl-modifications associated with RM defense systems of *D. pigrum* strains. White and light gray cells indicate that a modified motif was not detected or no SMRTseq data were available for a specific strain, respectively. Colored cells indicate that a motif was detected and the approximate genomic loci of the RM system responsible across strains are indicated by pink (R1H), blue (R2), or yellow (R3) cells. Sporadic occurrences of RM systems that do not appear conserved in more than a single strain are indicated by dark gray cells. (B) The organization of gene clusters within RM system integration site 1 hot spot (R1H), which harbors a diverse collection of RM systems, including type I (*n* = 2), type II (*n* = 7), and type III (*n* = 3), in addition to other mobile elements/transposons systems, including Hachiman, Gabija, and Kiwa defenses. R1H is flanked upstream by region containing genes for (p)ppGpp synthase/hydrolase and d-Tyr-tRNA (Tyr) deacylase proteins, and downstream by a region with genes for Y-family DNA polymerase and an rRNA pseudouridine synthase protein. Hypothetical genes are indicated by gray arrows labeled with an “H.”

In bacteria, individual RM systems can differ with respect to target sequence, active site architecture, and reaction mechanisms, but all recognize the methylation status of target sequences on incoming DNA and degrade inappropriately methylated (non-self) DNA. Type I to III systems largely recognize and digest a target sequence when it lacks the appropriate methyl group. In contrast, type IV systems, which lack a methyltransferase, are composed of a methyl-dependent restriction endonuclease (REase) that cuts a target sequence when it contains a specific methyl modification. RM systems and their recognition sequences are often strain specific. Therefore, we characterized and compared the repertoire of RM systems present in each of the 19 *D. pigrum* strains sequenced via SMRTseq, defining the methylome of each strain using SMRTseq kinetics (Basemod analysis) and predicting the recognition sequences of each system via REBASE analysis (71) (Fig. 7A; see also Table S4 and Text S1). Most of the modifications detected were m6A with only one m4C being found. There were several genes coding for m5C enzymes, but their products are not usually detected by the PacBio software. Only one positive m5C enzyme was identified. Among the RM systems, most were type II, although half the strains had a type IV enzyme of unknown specificity. The type I to III systems were associated with 19 individual target recognition motifs identified by methylome analysis (Fig. 7A; see also Table S4 and Text S1).

10.1128/mSystems.00425-21.4 TABLE S4 RM systems. Download Table S4, XLSX file, 0.02 MB. Copyright © 2021 Flores Ramos et al. 2021 Flores Ramos et al. https://creativecommons.org/licenses/by/4.0/ This content is distributed under the terms of the Creative Commons Attribution 4.0 International license.

### The *D. pigrum* type IV RM system is inversely related to a specific m5C-associated type II system.

We noted an inverse relationship between the presence of the *D. pigrum* type IV REase and a specific m5C-associated type II RM system that modified the second cytosine residue within the motif GCNGC (Fig. 7A). This inverse relationship was found to be interdependent between strains based upon a Fisher exact test (*P = *0.0055). The type II m5C system was present in nine *D. pigrum* genomes that lacked the type IV REase. Conversely, the type II m5C system was absent in eight strains that contained the type IV REase. Type IV REase that target m5C-modified motifs have the potential to limit the spread of RM systems that utilize m5C modifications. The *D. pigrum* type IV REase appears related (99% coverage/43% identity) to S. aureus SauUSI, a modified cytosine restriction system targeting S^5m^CNGS (either ^m5^C or ^5hm^C), where S is C or G. Based on the inverse relationship of the type IV and type II m5C systems, this strongly indicates that the *D. pigrum* type IV system targets m5C containing sequences, including GCNGC, GGNCC, and potentially the recognition sequence of the other m5C enzyme, M.Dpi3264ORF6935P.

### The type IV and specific m5C-associated type II RM systems are present at the same integration site.

To decipher the basis for the inverse relationship between these two RM systems, we sought to determine where each was incorporated in the *D. pigrum* genomes. In 18 of the 19 strains, the type IV REase or the m5C-associated type II system are inserted into the same genetic locus, dubbed R2 (Fig. 2B; see also Fig. S4A). In the one strain that carried both the type IV REase and the m5C-associated type II system, CDC4709-98, the type IV is present at R2, whereas the m5C system is integrated at an unrelated locus downstream from a tRNA-Leu site. MGEs that carry similar integrases tend to integrate at the same sites in the chromosome, but in most strains we did not observe any integrase or additional genes cooccurring with the RM systems at this site.

10.1128/mSystems.00425-21.9 FIG S4 The organization of GCs within RM system integration sites R2 and R3. (A) Organization of GCs within RM system integration site 2 (R2), which primarily encodes either a type II m5C-associated RM system or a type IV restriction system. R2 is flanked upstream by a region containing genes for a YacP-like NYN domain protein and an S8 family serine peptidase type protein, and downstream by genes for a DNA-3-methyladenine glycosylase protein, a hypothetical protein, and a copper-translocating P-type ATPase. (B) The organization of GCs within RM system integration site 3 (R3), which encodes two different type II RM systems. Arrows represent the direction of translation and the relative sizes of ORFs. Putative control proteins are highlighted in green; HsdR and HsdM are highlighted in red and blue, respectively; and fused hsdR/M ORFs are both red and blue. Proteins not identified as part of the RM system or those with currently unknown function are shown in gray. Download FIG S4, PDF file, 0.2 MB. Copyright © 2021 Flores Ramos et al. 2021 Flores Ramos et al. https://creativecommons.org/licenses/by/4.0/ This content is distributed under the terms of the Creative Commons Attribution 4.0 International license.

### Many *D. pigrum* RM systems compete for an integration hot spot.

Extending our analysis, we identified a genomic locus with an unexpectedly high frequency of variable genes across all 28 genomes. We dubbed this site RM system integration site 1 hot spot (R1H), because it harbors a diverse collection with 12 different RM systems spanning types I, II, and III across strains (Fig. 2B and Fig. 7B). Cooccurring with these RM systems in R1H, we also identified three of the antiphage deity-named defense systems: Hachiman, Gabija, and Kiwa present across seven strains (Fig. 7B). A third RM system integration site (R3) contained two different type II systems, along with an IS66 transposase family of genes (see Fig. S4B), consistent with the known association of defense systems and MGEs (72).

### *D. pigrum* encodes subtype II-A and I-E CRISPR-Cas systems.

**FIG 8:**
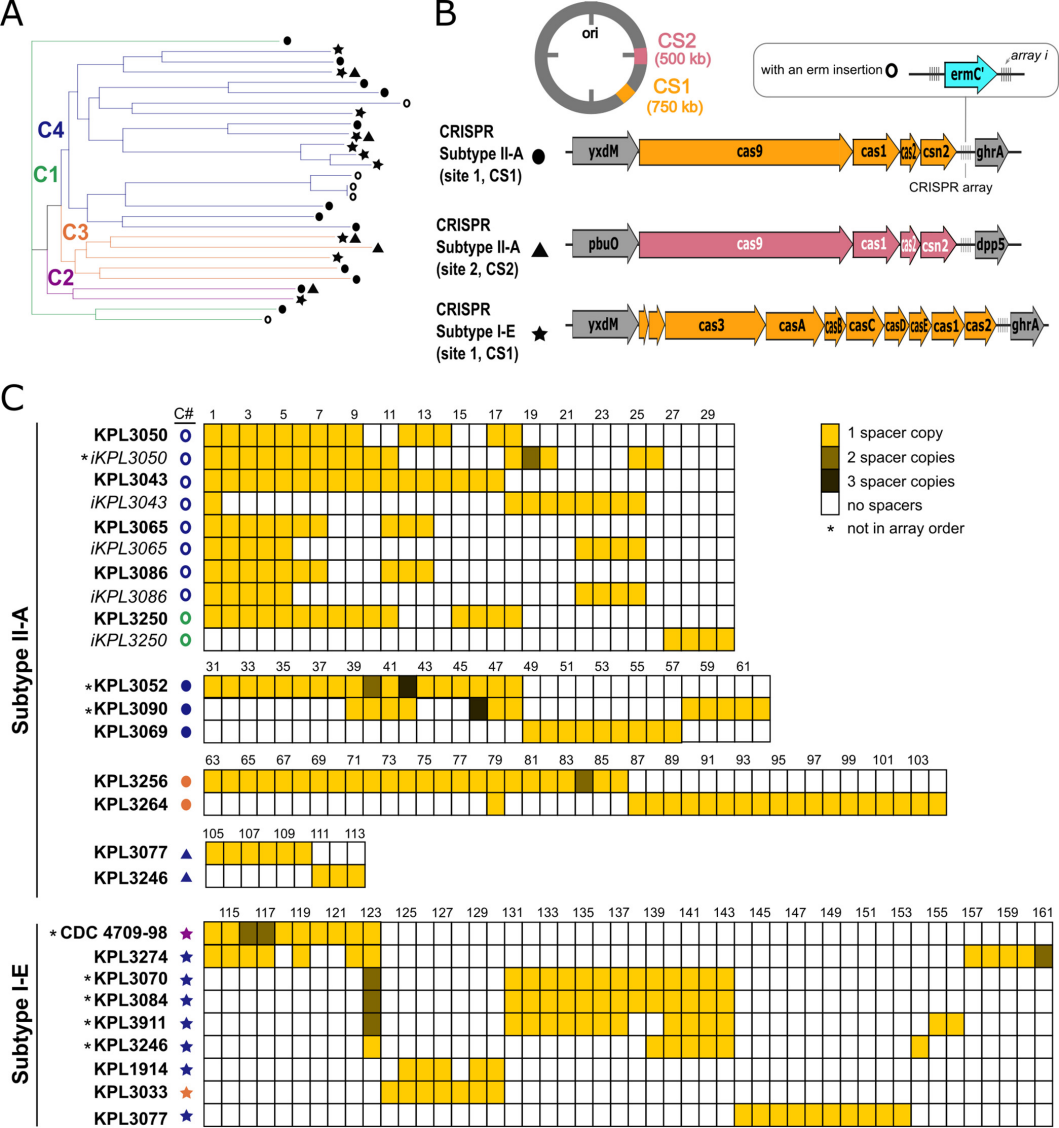
*D. pigrum* encodes subtype I-E and II-A CRISPR-Cas systems with a large but sparsely shared history of MGE invasion. (A) CRISPR-Cas subtype II-A (circles and triangles) and I-E systems (stars) were intermixed among strains in all four clades, with type II-A being most common (see Table S5A). Two distal clades had only a subtype II-A system (KPL3043, KPL3065/KPL3086, KPL3090, KPL3052, and KPL3069) or a subtype I-E system (KPL3070, KPL3084, and KPL391). Three genomes (KPL3077, KPL3246, and CDC2949-98) have both types of system, with each at a different locus. (B) The most common location, CRISPR-Cas system insertion site (CS1), is between the ABC transporter permease protein (*yxdM*) and the glyxyolytate/hydroxypyruvate reductase A (*ghrA*) genes. However, subtype II-A systems are also found in between the guanine/hydoxanthine permease (*pbuO;* NCS2 family permease) and dipeptidyl-peptidase 5 (*dpp5;* S9 family peptidase) genes at CRISPR-Cas insertion site 2 (CS2). Five of the strains with a subtype II-A system in CS1 had a predicted rRNA adenine *N*-6-methyltransferase (*ermC′*) gene integrated in their CRISPR arrays (open circles) (C) Representation of the spacers (see Table S5B and Text S1 in the supplemental material) found among the different CRIPSR systems in the 19 closed genomes. We found 161 unique spacers, less than one-third of which were homologous to phages and plasmids found among other *Firmicutes*. Strains KPL3050, KPL3250, KPL3065/KP3086, and KPL3043 shared the most spacers among the subtype II-A CRISPR-Cas system, with the distal clade of KPL3043 and KPL3065/KPL3086 sharing 15 spacers. The distal clade with KPL3070, 3084, and 3911 shared the most spacers (12) among the subtype I-E system. CRISPR-Cas systems and spacers hits were determined using the CRISPRdetect and CRISPRtarget database on 16 February 2019, while shared spacers were determined using CRIPSRCompar on 18 March 2019.

CRISPR-Cas systems provide adaptive/acquired defense (immunity) against MGEs (46). All of the complete *D. pigrum* genomes encoded at least one subtype II-A or I-E CRISPR-Cas system (Fig. 8A; see also Table S5A), based on the CRISPRDetect database (73). Of the 32 CRISPR-Cas systems detected, 22 are subtype II-A, which is mostly found in *Firmicutes* (74) and is the predominant CRISPR-Cas system among *Lactobacillus* spp. (75). Subtypes II-A (circles, Fig. 8A) and I-E (stars, Fig. 8A) CRISPR-Cas systems were generally intermixed within the four major clades, although several distal clades harbored only one type. A single genomic locus (CS1) contained either a subtype II-A or a subtype I-E CRISPR-Cas system in all 19 closed genomes (Fig. 2B and Fig. 8A and B). A second CRISPR-Cas system (triangles, Fig. 8A) was found at a second location (CS2) in 4 of these 19 genomes, from three of the four clades (Fig. 8B).

10.1128/mSystems.00425-21.5 TABLE S5 CRISPR-Cas systems. Download Table S5, XLSX file, 0.02 MB. Copyright © 2021 Flores Ramos et al. 2021 Flores Ramos et al. https://creativecommons.org/licenses/by/4.0/ This content is distributed under the terms of the Creative Commons Attribution 4.0 International license.

### *D. pigrum* CRISPR-Cas spacers point to undiscovered *D. pigrum* MGEs.

Each of the 19 closed genomes included at least one complete CRISPR array. (As expected, most of the arrays were incomplete in the unclosed genomes.) Examining the CRISPR arrays in the 19 closed genomes revealed two key findings. First, the spacer sequences predict the existence of a diversity of undiscovered *D. pigrum* phages and plasmids with mean numbers of spacers per array of 13 (median, 12.5) for subtype II-A and 11.1 (median, 12) for subtype I-E (see Table S5A). Second, spacer sequences show a sparsely shared history of exposure to many MGEs (Fig. 8C; see also Table S5). Only 60 of the 161 unique identified spacers were shared by more than one strain (Fig. 8C). The exceptions to this limited shared history were two distal clades with shorter branch lengths within Clade 4, which shared 15 and 12 spacers, respectively. Of these 27 spacers, 9 had similarity to known MGEs (see Text S1). A few other shared spacers were scattered among *D. pigrum* strains outside these two distal clades. For example, *D. pigrum* KPL3033 (clade 3) and KPL1914 (clade 4) shared five spacers (Fig. 8C), one of which matched to the *Clostridium* phiCDHM19 phage (LK985322; spacer 129) (76). These shared spacers suggest strains within the host-range of specific MGEs. Spacer similarity to known MGEs indicated prior *D. pigrum* exposure to phage and plasmid elements that might be related to those found in other genera of *Firmicutes*, e.g., *Clostridium*, *Lactococcus*, Streptococcus, Staphylococcus, and *Enterococcus*. However, only 46/161 spacers had significant matches (match score >15) to previously identified MGEs and none had matches to any of the predicted prophage from Fig. 5, indicating that that *D. pigrum* CRISPR-Cas systems likely target a variety of yet-to-be-identified host-specific *D. pigrum* plasmids and phages.

## DISCUSSION

Multiple recent studies of the composition of human nasal microbiota identify *D. pigrum* as a candidate beneficial bacterium (1–30). Our systematic analysis of 28 *D. pigrum* strain genomes, including 19 complete and closed genomes, reveals a phylogeny in which strains collected 20 years apart intermingled in clades and showed remarkable stability in genome structure (Fig. 1 and 2). Many of the older *D. pigrum* strains were collected in the context of human disease (48), making it unclear whether these strains were contributors to disease, bystanders, or contaminants. In a previous analysis, we detected no virulence factors in the genomes of nine of these older strains from Laclaire and Facklam (48), consistent with *D. pigrum* having a commensal or mutualistic relationship with humans (28). Adding further support for this, we detected no virulence factors in any of our newer strains here (see Text S1), which were all isolated from healthy volunteers. Plus, many of the older strains are closely related in the phylogeny with these recent healthy-donor-derived strains (Fig. 1). These findings are consistent with there being only a few isolated reports of *D. pigrum* growth in samples from different types of infections (77–82). Of these, the repeated detection of *D. pigrum* alone in keratitis/keratoconjunctivitis raises the possibility that some strains might be rare causes of eye surface infection (83–86). We recommend future genome sequencing of ophthalmic infection isolates to ascertain whether and how these vary from currently sequenced avirulent strains.

Our results show that strain-level variation in *D. pigrum* is driven by gene gain/loss in variable regions located between large blocks of syntenic DNA (Fig. 2). This pattern is consistent with the findings of Oliveira et al. for the chromosomal structures of 80 different bacterial species (87). Furthermore, *D. pigrum* core GCs exhibit very high conservation of nucleotide sequences (≥97.5%), and the 19 closed genomes show the order of syntenic blocks of core genes is conserved (Fig. 2A). *D. pigrum* has an average genome size of 1.93 Mb (median, 1.91 Mb) (see Table S1) with an open pangenome (Fig. 3B). About 64% of each *D. pigrum* strain genome consists of core CDS, whereas only about 30% of the *D. pigrum* pangenome consists of core GCs. This is similar to the percentage of core genes in the pangenomes of other colonizers of the human upper respiratory tract, such as Staphylococcus aureus (36%) and Streptococcus pyogenes (37%) (88).

HGT, much of it likely mediated by MGEs, plays an important role in strain diversification in free-living bacteria. However, a systematic search identified few such elements per genome among *D. pigrum* strains. In terms of MGEs that commonly mediate HGT, we detected no autonomous plasmids. However, we identified one complete and three partial predicted prophages (Fig. 5) among 27 distinct strain genomes (2 of the 28 genomes were almost identical). To our knowledge, the predicted complete prophage (L1) is the first phage element identified in *D. pigrum*. The disparate nature of these candidate prophages compared to those in current databases is consistent with *D. pigrum* having its own specific pool of yet-to-be-identified phage predators, consistent with the strain-level specificity of many known phages. This is further supported by the scarce homology of the phage spacers in the CRISPR arrays to those available in the databases. However, some *D. pigrum* prophages might share a distant common ancestor with streptococcal phages, as almost one fifth L1’s and at least one third of L2’s (77/202) and L3’s (51/187) predicted genes shared the most similarities to Streptococcus phage genes (see Text S1). Based on our findings, we predict that phage elements targeting *D. pigrum* have a narrow host range, consistent with patterns exhibited by other *Firmicutes*-targeting phages, such as those targeting *Listeria* and Clostridium difficile (58, 76). The identification of *D. pigrum* prophages creates the opportunity for future work to systemically query nasal metagenomic data sets for these and other *D. pigrum* MGEs, as well as for CRISPR spacers.

In terms of MGEs that commonly move within genomes, *D. pigrum* genomes host a group II intron and most also host a small number of predicted transposases and/or integrases (see Table S3). Once present in a genome, IS movement can lead to phenotypic variation among closely related strains through disruption of open reading frames (ORFs) or changes in transcription due to insertion in or adjacent to promoters (65).

The small number of MGEs identified might be related to the multiple defense mechanisms present in each *D. pigrum* genome. RM systems are ubiquitous in bacteria and present in ∼90% of genomes (71). They play a key role in protecting bacterial genomes from HGT, including MGEs, and maintaining genome stability. The variety of RM systems within and among *D. pigrum* genomes is consistent with this role. To our knowledge, this is the first report of a strongly inverse relationship between an m5C-targeting type IV REase and an m5C-associated type II system within the same chromosomal locus. A similar relationship was described previously for two antagonistic type II systems in Streptococcus pneumoniae, where strains possess either DpnI (which cleaves only modified G^m6^ATC) or DpnII (which cleaves only unmodified GATC) (89). It remains unclear whether the inverse relationship observed between the two *D. pigrum* systems results from competition for an integration site within a *D. pigrum* genome (R2; Fig. 7) or whether the type II system’s m5C-modified target motif is incompatible with the type IV REase. Determination of the exact underlying mechanism for this type IV/type II relationship warrants future investigation and has implications for other bacterial genomes.

CRISPR-Cas systems are another common bacterial defense system that maintain genomic stability. In a recent analysis of complete genomes from 4010 bacterial species in NCBI RefSeq, 39% encode *cas* clusters (74). Several characteristics of the predicted *D. pigrum* CRISPR-Cas systems suggest these are active. First, the preservation of repeats and spacers along with all of the core Cas gene suggests active systems, since inactive systems often show evidence of degeneration in terms of inconsistent repeat/spacer lengths (75). Second, the diversity of spacers among *D. pigrum* strains supports the likelihood of activity (90). *D. pigrum* belongs to the order *Lactobacillales* in the phylum *Firmicutes*. Similar to our observations in *D. pigrum* (Fig. 8), among 171 *Lactobacillus* species, when multiple CRISPR-Cas systems are present in a single genome these are most often a subtype I-E and subtype II-A, and these two subtypes predominate among type I and II systems in *Lactobacillus* (75). More broadly, there is a positive association between subtype I-E and subtype II-A systems within the phylum *Firmicutes* (74). Within *Lactobacillus*, type I systems contain the longest arrays (average 27 spacers) (75), and we see something similar among the *D. pigrum* strains. Of the spacers with matches to known plasmid and phage elements in the GenBank-Phage, Refseq-Plasmid, and IMGVR databases in CRISPRTarget, almost half of the identified spacers corresponded to plasmid elements. Subtype II-A systems in *Lactobacillus* actively transcribe and encode spacers that provide resistance against plasmid uptake based on plasmid interference assays in which an exogenous plasmid is engineered to contain endogenous spacer sequences (75, 91). This defense mechanism might explain the lack of autonomous plasmids in *D. pigrum* strain genomes to date.

The majority of *D. pigrum* CRISPR spacers lack homology to known MGEs. This is consistent with a large-scale analysis of bacterial and archaeal genomes in which only 1% to 19% of spacers (global average ∼7%) in genomes match known MGEs, mostly phages and plasmids and uncommonly to self. Also, spacers without a match share basic sequence properties with MGE-matching spacers pointing to species-specific MGEs as the source for CRISPR spacers (92). In this context, our findings indicate *D. pigrum* strains defend themselves against a wealth of yet-to-be-identified *D. pigrum-*specific MGEs. Some of these MGEs might be key to developing a system for genetic engineering of *D. pigrum*.

Like other pangenomic studies, this one has both general and species-specific limitations. First, the open pangenome indicates that the accessory gene space of *D. pigrum* remains to be more completely assessed through sequencing strains beyond the 28 investigated here. All but 1 of these 28 strains were collected in North America, so a next step is genome sequencing *D. pigrum* isolates from human volunteers from diverse geographic settings on other continents. Second, many more isolates would need to be collected over time to generate a comprehensive analysis of *D. pigrum* strain circulation in humans across the United States, and beyond. Third, this is a systematic computational prediction of genome defense systems and MGEs. The next step is experimental verification of the function of these computationally predicted entities, which underscores the need for a system to genetically engineer *D. pigrum*. Fourth, in this study, we systematically identified known genomic elements that can affect bacterial genomic stability. This leaves a large proportion of *D. pigrum*’s accessory genome to be explored in future work.

In conclusion, a growing number of studies point to *D. pigrum* as a candidate beneficial bacterium with the potential for future therapeutic use to manage the composition of human nasal microbiota to prevent disease and promote health (40). One standard for bacterial strains for use in humans, either in foods, the food chain or therapeutics, is the absence of antimicrobial resistance (AMR) genes against clinically useful antibiotics (93). A prior report of 27 *D. pigrum* strains shows all are susceptible to clinically used antibiotics with the exception of frequent resistance to erythromycin (48). Consistent with this, only 6 of the 17 new *D. pigrum* genomes reported here encode AMR genes with predicted resistance to erythromycin and/or kanamycin (see Text S1). This confirms the broad antimicrobial susceptibility of *D. pigrum*. Further supporting its safety, we detected no virulence factors in these 28 genomes. Moreover, this pangenomic analysis of 28 *D. pigrum* isolates collected over the span of 20 years revealed remarkable stability in both strain circulation and chromosomal structure. Consistent with this stability, we detected relatively few MGEs in each genome; however, each genome hosted a variety of defense systems for protection against MGEs, and HGT in general. The antibiotic susceptibility, genomic stability, capacity for defense against HGT, and lack of known virulence factors described here all support the safety of *D. pigrum* as a candidate for use in clinical trials to determine its potential for therapeutic use.

## MATERIALS AND METHODS

### Collection of new *D. pigrum* strains.

We collected strains of *D. pigrum* from children and adults using supervised self-sampling of the nostrils with sterile swabs at scientific outreach events in Massachusetts in April 2017 and April 2018 under a protocol approved by the Forsyth Institutional Review Board (FIRB 17-02). All adults provided informed consent. A parent/guardian provided informed consent for children (<18 years old), and all children ≥5 years provided assent. (Self-sampling by children was considered evidence of assent.) Briefly, participants rubbed a sterile rayon swab (BBL, Franklin Lakes, NJ) around the surface of one nasal vestibule (nostril) for 20 s, and then we immediately inoculated this onto BBL Columbia colistin-nalidixic acid agar with 5% sheep’s blood (CNA blood agar). After 48 h of incubation at 37°C in a 5% CO_2_ enriched atmosphere, each CNA blood agar plate was examined, and colonies with a morphology typical for *D. pigrum* were selected for purification. Purified isolates were verified to be *D. pigrum* by 16S rRNA gene colony PCR (GoTaq Green; Promega, Madison, WI) using the primers 27F and 1492R and Sanger sequencing from primer 27F (Macrogen USA, Cambridge, MA).

### Genomic DNA extraction.

All *D. pigrum* strains were cultured from frozen stocks on CNA blood agar plates at 37°C with 5% CO_2_ for 48 h. For each strain, cells from eight plates were harvested with a sterile cotton swab (Puritan, Guilford, ME) and resuspended in 1 ml of sterile 1× phosphate-buffered saline (PBS; Fisher, Waltham, MA). Then, 10 100-μl resuspensions were spread and grown on 47-mm, 0.22-μm-pore-size polycarbonate membranes (EMD Millipore, Burlington, MA) atop CNA blood agar plates at 37°C with 5% CO_2_ for 24 h. Three membranes were resuspended in 20 ml of TES buffer (20 mM Tris-HCl, 1 M [pH 8.0]; 50 mM EDTA; filter sterilized) and normalized to an optical density at 600 nm of 1.0 ± 0.02. Half the resuspension was spun down at 5,000 rpm (2,935 × *g*) for 10 min at 4°C. The genomic DNA was extracted using a Lucigen Masterpure (Epicentre, Middleton, WI) Gram-positive DNA purification kit according to the manufacturer’s instructions with the following modifications: we increased the amount of Ready-Lyse lysozyme added per preparation to 2.5 μl and deleted the bead-beating step. The extracted genomic DNA was assessed for quantity using a Qubit per manufacturer instructions, for quality on a 0.5% agarose gel, and for purity by measuring 260/280 and 260/230 ratios on a NanoDrop spectrophotometer.

### Whole-genome sequencing, assembly, and annotation.

Single molecule, real-time sequencing (SMRTseq) was carried out on a PacBio Sequel Instrument (Pacific Biosciences; Menlo Park, CA) with V2.1 chemistry, following standard SMRTbell template preparation protocols for base modification detection. Genomic DNA samples (5 to 10 μg) were sheared to an average size of 20 kbp via G-tube (Covaris, Woburn, MA), end repaired, and ligated to hairpin barcoded adapters prior to sequencing. Sequencing reads were processed using the Pacific Biosciences SMRTlink pipeline (https://smrtflow.readthedocs.io/en/latest/smrtlink_system_high_level_arch.html) according to the HGAP version 4.0 assembly tool standard protocol. Single contigs generated through HGAP were also processed through Circlator version 1.5.5 using default settings to assign the start site of each sequence to *dnaA* (94). All genomes were annotated with the NCBI’s Prokaryotic Genome Annotation Pipeline (PGAP) (95, 96) and uploaded to the NCBI database (accession numbers CP040408 to CP040424).

### Determination of the conservative core genome and the pangenome sizes.

All the genomes were annotated with Prokka version 1.13.0 (97) prior to identification of the conservative core genome with GET_HOMOLOGUES version 3.1.4. (42, 98) using the cluster intersection (compare_clusters.pl; blastp) result of three algorithms: bidirectional best-hits (BDBH), cluster of orthologs (COG) triangles (99), and Markov Cluster Algorithm OrthoMCL (OMCL) (100). The nucleotide level clustering for each of these algorithms was calculated with the get_homologues.pl script and the following parameters: -a CDS, -A, -t 28, -c, -R, and either -G for COG, -M for OMCL, or no flag for BDBH. To obtain the nucleotide instead of the protein outputs, blastn instead of blastp was used to report clusters (parameter –a CDS).

The pangenome was established using the OMCL and COG triangle algorithm with –t 0 parameter to get all possible clusters when running get_homologues.pl. The total clusters from the OMCL and COG pangenomes were then used by compare_clusters.pl with the –m flag to create a pangenome matrix tab file. The cloud, shell, soft core, and core genome of the isolates were then determined using the parse_pangenome_matrix.pl script in GET_HOMOLOGUES using the -s flag and the pangenome matrix tab file. The average nucleotide identity and genome composition analysis were also implemented (using the –A and –c parameters, respectively, in get_homologues.pl). For the genome composition analysis, which shows how many new CDS are added to the pangenome per new genome addition, the conservative default parameters and a random seed (–R) of 1234 was selected.

### Phylogenomic tree construction.

A core gene alignment was created for phylogenetic analysis using the nucleotide sequences from the conservative single-copy core GCs (*n* = 1,102) identified with GET_HOMOLOGUES. These GCs were aligned with MAFFT version 7.245 (101) using default settings, renamed to match the isolate’s strain name, and concatenated into an MSA file through the catfasta2phyml.pl script using the concatenate (–concatenate) and fasta (-f) parameters (copyright 2010-2018 Johan Nylander). The core gene multiple sequence alignment was converted into a phylip file format with Seaview version 4.7 (102). An unrooted phylogenetic tree of the conservative single-copy core (Fig. 1) was generated using this phylip file and IQ-Tree version 1.6.9. (103). The ModelFinder function in IQ-Tree identified the GTR+F+ as the appropriate substitution model for tree construction (BIC value 5597954.8128) (104). Using this model, 553 maximum-likelihood searches with 1,000 ultrafast rapid Bootstraps (105) were used to generate the final maximum likelihood tree (ML = −2854949.911). A clade was defined as a monophyletic group of strains sharing a well-supported ancestral node. SNP pairwise distance in the rooted and unrooted tree were determined using the “harrietr” R package (https://cran.r-project.org/web/packages/harrietr/README.html) applied with an in-house script. Pairwise MASH-distances were calculated for all *D. pigrum* strains using the implementation of the MASH algorithm (106) in the PanACoTA pipeline (107). Code and data files for this part of the analysis are available online (https://github.com/KLemonLab/DpiMGE_Manuscript/blob/master/SupplementalMethods_PhylogeneticDistances.md).

### Synteny analysis.

We performed a whole-genome sequence alignment on all closed genomes using progressive Mauve in Mauve version 2.4.0.r4736 with its default settings (50, 51). For the five genomes that we were unable to circularize, we manually fixed the start site to *dnaA* and added NNNNNNNNN to the region concatenating the ends of the contigs to mark it as a region of uncertainty in the synteny alignment. Manual curating was done with SnapGene version 4.2.11 GUI platform (SnapGene software from GSL Biotech).

### Functional analysis of the pangenome using Anvi’o.

All genomes were reannotated with an updated Prokka version (1.14.6) (97) with default parameters, including gene recognition and translation initiation site identification with Prodigal (108). The pangenome was analyzed using Anvi’o version 7 (52, 53). We followed the pangenome workflow to import Prokka annotated genomes into Anvi’o (http://merenlab.org/2017/05/18/working-with-prokka/), followed by the addition of functional COG annotations using the anvi-run-ncbi-cogs program with the –sensitive flag (runs sensitive version of DIAMOND [109]) and the 2020 updated COG20 database (110, 111). KEGG/KOfam (112, 113), and Pfam (114) annotations were also added to each genome .db file, as well as hmm-hits (115). The pangenome was calculated with the anvi-pan-genome program (flags: –minbit 0.5, –mcl-inflation 10, and –use-ncbi-blast) using blastp search (116), muscle alignment (117), “minbit heuristic” (118) to filter weak hits, and the MCL algorithm (119). The functional and geometric homogeneity index, and the rest of the information shown in Fig. S3D were calculated following the standard Anvi’o pangenomic pipeline (http://merenlab.org/2016/11/08/pangenomics-v2). The core (*n* = 28), soft core (28 > *n* ≥ 26), shell (26 > *n* ≥ 3), and cloud (*n* ≤ 2) annotations from GET_HOMOLOGUES were added to the Anvi’o pangenomic database using the interactive interface. We defined the accessory as GCs present in ≤25 genomes and core as GCs present in ≥26 genomes. The output of this Anvi’o pangenomic analysis and the code used to generate it are available online (https://github.com/KLemonLab/DpiMGE_Manuscript/blob/master/SupplementalMethods_Anvio.md). We used the summary file we exported from the Anvi’o pangenomic analysis to generate the functional enrichment plots shown in Fig. 4 and in Fig. S3E and F using an in-house R script (https://github.com/KLemonLab/DpiMGE_Manuscript/blob/master/SupplementalMethods_COGs.md) to wrangle and extract information on the informative COG20 annotated gene clusters (120, 121).

### PPanGGOLiN analysis.

Gene clustering and annotation data were exported from the Anvi’o output and imported into PPanGGOLiN version 1.1.141 (Partitioned PanGenome Graph Of Linked Neighbors) (68) to create a partitioned pangenome graph (PPG) that assigned GCs to the “persistent,” “shell,” and “cloud” partitions. Regions of genome plasticity (RGPs) and spots of insertion were predicted (122), and subgraphs of the hot spots of interest were generated by providing the sequence of the flanking proteins in a fasta file. The output of the PPanGGOLiN analysis and the code used to generate it are available online (https://github.com/KLemonLab/DpiMGE_Manuscript/blob/master/SupplementalMethods_PPanGGOLiN.md). The subgraphs represented as inserts on Fig. 2B were obtained with the command “ppanggolin align -p pangenome.h5 –getinfo –draw_related –proteins” using the amino acid sequences for the proteins upstream and downstream of each spot of interest. Since PPanGGOLiN does not currently allow creation of subgraphs using GCs imported from external clustering methods, the pangenome was run again using the default PPanGGOLiN workflow with MMseqs2 clustering (default settings: –identity 0.8, –coverage 0.8, and –defrag).

### Characterization of MGEs.

We searched all genomes for phage elements using the PHASTER database and web server (http://phaster.ca) on 8 November 2018 (56, 57). We took the “intact” phage elements as defined by a phage score of >90 and queried their ORFs using blastp to manually reannotate their phage genes in the SnapGene GUI.

We searched for plasmid elements in all genomes using the PlasmidFinder 2.0 database and GUI interface (https://cge.cbs.dtu.dk/services/PlasmidFinder/) on 13 November 2018 using the default parameters (62). For strains with hits for a plasmid element, ORFs 1,000 kb upstream and downstream of the element were queried through blastp. Manual gene reannotation was performed using the SnapGene GUI platform.

The summary file exported from the Anvi’o pangenomic analysis (see above) was also used for the identification of MGEs on the Prokka, COG20, Pfam, and KOfam annotations. We identified 23 GCs as coding for putative transposases. GC alignments were visually inspected in AliView (123), and full-length representative sequences were selected for Pfam search at the Pfam batch sequence search/HMMER website (114, 124). We identified eight GCs with complete (≥80% coverage) Pfam Transposase (tnp) domains as true predicted transposases and five GCs with complete (≥80% coverage) Pfam rve domains as integrases. We used Operon ConTextulization Across Prokaryotes to Uncover Synteny (OCTAPUS; https://github.com/FredHutch/octapus) to identify the gene neighborhoods in which the selected transposases and integrases were located across all 28 *D. pigrum* genomes (see Table S3). The approach used by OCTAPUS is to search for a set of defined query genes across a collection of reference genomes by translated amino acid alignment and then to summarize the results by their physical colocation and organization. In this way, operon structure can be identified as the consistent colocation of a set of genes across multiple genomes in the same relative orientation (including both position and strand). The groups of genes identified with OCTAPUS at minimum percent identity 85% and minimum coverage 80% were visualized using clinker (https://github.com/gamcil/clinker) (125), and summary data provided in (see Table S3) were calculated using the matrixStats package (https://github.com/HenrikBengtsson/matrixStats). Detailed methods for this part of the analysis, as well as relevant files, are available online (https://github.com/KLemonLab/DpiMGE_Manuscript/blob/master/SupplementalMethods_MGEs.md).

We similarly used OCTAPUS to identify the gene neighborhood of the group II intron identified with Anvi’o and PPanGGOLiN (GC_00000001). Using Pfam, we confirmed two predicted domains in a sequence from *D. pigrum* KPL3250 in GC_00000001: a reverse transcriptase and a maturase. The best hit in a blastx search with this same sequence against the Bacterial Group II Intron Database was to the bacterial class C intron-encoded protein from La.re.I1 in Lactobacillus reuteri with 44% identity and 65% similarity over 419 amino acids (70).

### Base modification analysis and prediction of restriction-modification systems.

For methylome analysis, interpulse durations were measured and processed for all pulses aligned to each position in the reference sequence. We used Pacific Biosciences’ SMRTanalysis v8, which uses an *in silico* kinetic reference and a t-te st-based kinetic score detection of modified base positions, to identify modified positions (126).

We identified RM systems using SMRTseq data, as previously described (127), using the SEQWARE computer resource, a BLAST-based software module in combination with the curated restriction enzyme database (REBASE; http://rebase.neb.com/rebase/rebase.html) (71). Prediction was supported by sequence similarity, presence, and order of predictive functional motifs, plus the known genomic context and characteristics of empirically characterized RM system genes within REBASE. This facilitated reliable assignment of candidate methyltransferase genes to each modified motif based on their RM type.

### Detection of 5-methylcytosine.

For *D. pigrum* CDC4709-98 (aka KPL1934), the presence of 5-methylcytosine in the predicted methylation motif GCNGC was assessed as previously described (127). Briefly, gDNA harvested with a Masterpure Complete DNA/RNA purification kit was bisulfite treated using an EpiMark bisulfite conversion kit (NEB, Ipswich, MA)—both according to manufacturer’s instructions, except for a final elution volume of 20 μl in the EpiMark kit. We then selected two genomic regions: each ≤700 bp containing ≥4 GCNGC motifs. We PCR amplified each region from 1 μl of the converted gDNA using TaKaRa EpiTaq HS for bisulfite-treated DNA (TaKaRa Bio USA, Mountain View, CA) according to the manufacturer’s instructions with the primers designed by MethPrimer: oKL732 (5′-AAGTTTATTTTTTTGAGTTTGTTG-3′), oKL733 (5′-TACCCATAAAATTATCACCTTC-3′), oKL734 (5′-ATTGATTTAGTAATTTTTTTGGAATAT-3′), and oKL735 (5′-TAAATAACTCTACAAAAAACTCAACTTACC-3′). After amplicon purification with a QIAquick PCR purification kit (final elution, 40 μl; Qiagen; Germantown, MD), we used Sanger sequencing (Macrogen, USA) of each PCR product to detect cytosine methylation within the predicted motif. Additional m5C-based modified motif analysis was carried out for Dolosigranulum pigrum KPL3250 using MFRE-Seq, as previously described (128).

### Prediction of CRISPR-Cas systems.

CRISPR cas genes were detected using the CRISPRFinder (https://crispr.i2bc.paris-saclay.fr/Server/) (129), and the array elements downstream from these genes were found using CRISPRDetect software (http://crispr.otago.ac.nz/CRISPRDetect/predict_crispr_array.html) (73). The spacers identified using CRISPRDetect were queried through databases of possible phage targets in the GenBank-Phage, Refseq-Plasmid, and IMGVR databases with CRISPRtarget (http://crispr.otago.ac.nz/CRISPRTarget/crispr_analysis.html) (73, 130), keeping hits with a cutoff score greater than 14. These spacers were also queried against the predicted L1 to L4 prophages through CRISPRtarget using a cutoff score >0. All gene and array element searches were completed on the webserver on 16 February 2019—with the exception of the spacers’ query through the L1 to L4 prophages on 27 July 2021—using the default parameters. We also queried the genomes through CRISPRdb and CRISPRCompar (https://crispr.i2bc.paris-saclay.fr) website on 18 March 2019 to identify and annotate spacers shared among the different strains, keeping hits with scores higher than 15 to indicate similarity (129, 131, 132).

### Data availability.

All genomes are available from the NCBI. Table 1 lists the accession numbers for each *D. pigrum* strain genome used in this study.
