# Supplementary material for: Genomic Stability and Genetic Defense Systems in Dolosigranulum pigrum, a Candidate Beneficial Bacterium from the Human Microbiome
Source: mSystems. 2021 Sep 21;6(5):e00425-21. doi: 10.1128/mSystems.00425-21 (PMC8547433; doi:10.1128/mSystems.00425-21)
Supplement: TABLE S1 [file msystems.00425-21-st001.pdf]

Table S1. Pangenome contribution of all 28 *D. pigrum* genomes[a]

Strain name	Size (bp)	GC%	CDS	tRNA	rRNA	tmRNA	Core	Soft core	Shell	Cloud
ATCC 51524	1,862,135	39.57%	1684	23	9	1	1134	195	245	42
KPL1914	1,890,316	39.53%	1770	53	12	1	1134	197	293	52
CDC 39-95	1,859,258	39.72%	1708	52	6	1	1134	192	250	68
CDC 2949-98	1,886,398	39.57%	1693	53	6	1	1134	192	245	52
CDC 4294-98	2,014,679	39.47%	1914	55	5	1	1134	190	310	211
CDC 4420-98	1,934,436	39.65%	1776	55	4	1	1134	197	286	84
CDC 4545-98	1,861,299	39.58%	1704	53	4	1	1134	181	245	74
CDC 4709-98	1,987,788	39.68%	1778	54	12	2	1134	197	290	37
CDC 4199-99	1,976,602	39.65%	1795	56	7	1	1134	165	311	105
CDC 4791-99	1,873,869	39.60%	1692	54	5	1	1134	190	238	62
CDC 4792-99	1,893,917	39.43%	1734	56	6	1	1134	194	248	87
KPL3033	1,958,196	39.55%	1801	53	12	1	1134	196	321	72
KPL3043	1,885,610	39.80%	1714	53	12	1	1134	194	274	21
KPL3050	2,043,806	39.30%	1840	53	12	1	1134	193	339	72
KPL3052	2,001,464	39.43%	1820	53	12	1	1134	194	342	68
KPL3065	1,878,426	39.82%	1694	53	12	1	1134	197	278	13
KPL3069	1,977,296	39.58%	1870	53	12	1	1134	177	347	123
KPL3070	1,883,862	39.76%	1738	53	12	1	1134	192	292	36
KPL3077	1,956,924	39.23%	1838	53	12	1	1134	194	352	67
KPL3084	1,906,822	39.61%	1757	54	12	1	1134	193	302	36
KPL3086	1,864,691	39.79%	1706	53	12	1	1134	192	278	31
KPL3090	2,009,643	39.74%	1891	56	12	1	1134	193	310	127
KPL3246	1,946,134	39.79%	1720	54	12	1	1134	192	276	44
KPL3250	1,904,333	39.61%	1714	54	12	1	1134	196	263	44
KPL3256	1,941,301	39.73%	1800	54	12	1	1134	193	307	65
KPL3264	1,984,655	39.70%	1811	53	12	1	1134	186	323	86
KPL3274	1,875,895	39.76%	1684	53	12	1	1134	188	239	51
KPL3911	1,900,145	39.59%	1772	54	12	1	1134	193	304	44
**AVERAGE**	**1927139**	**39.62%**	**1765**	**53**	**10**	**1**	**n.a.**	**191**	**290**	**67**
**MEDIAN**	**1905578**	**39.61%**	**1764**	**53**	**12**	**1**	**n.a.**	**193**	**291**	**64**

[a]With addition of new strain genomes, the average genome size increased from 1.86 Mb (median 1.88 Mb) (28) to 1.93 Mb (median 1.91 Mb), while the GC content remained at 39.6%. The CDS, tRNA, rRNA, and tmRNA are based on the Prokka::prodigal annotations. The average length of the CDS was 950 bp, while the average number of genes per kb was 0.916. There was also an increase in the number of predicted coding sequences from 1750 (median 1734) (28) to 1765 (median 1764). The predicted tRNA, rRNA, and tmRNA sequences averaged to 53, 10, and 1, respectively.
