# Supplementary figures and images for: Genomic Stability and Genetic Defense Systems in Dolosigranulum pigrum, a Candidate Beneficial Bacterium from the Human Microbiome
Source: mSystems. 2021 Sep 21;6(5):e00425-21. doi: 10.1128/mSystems.00425-21 (PMC8547433; doi:10.1128/mSystems.00425-21)

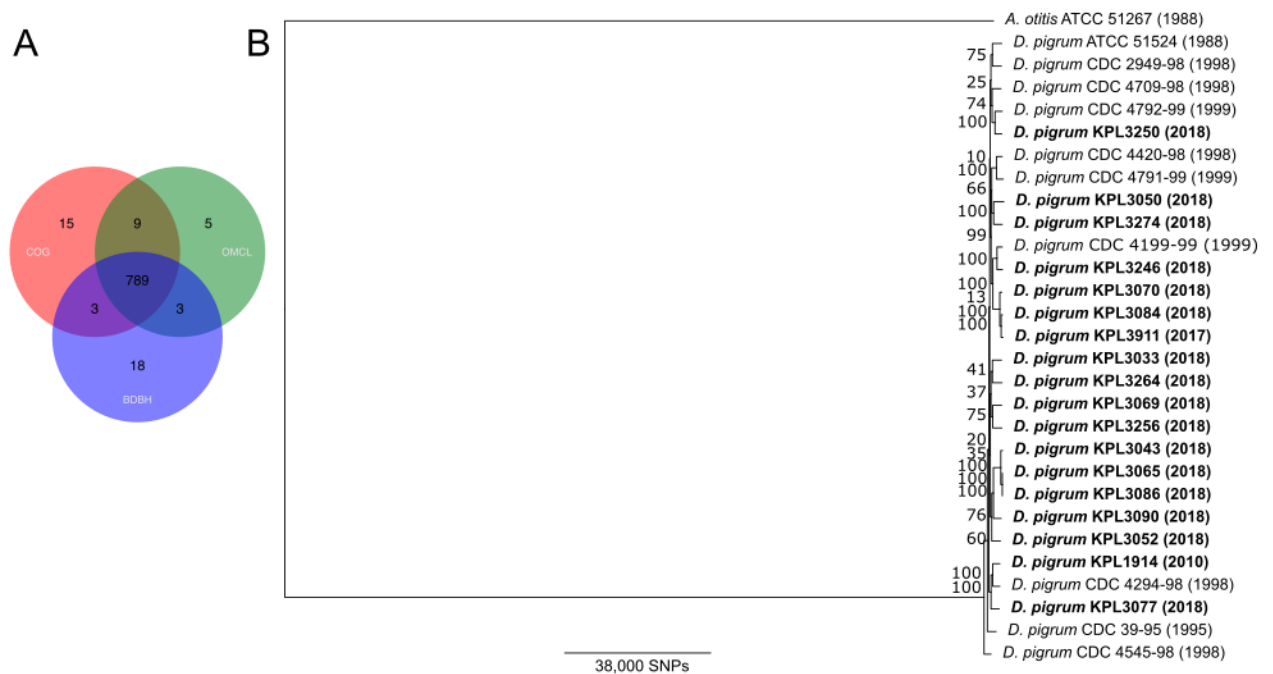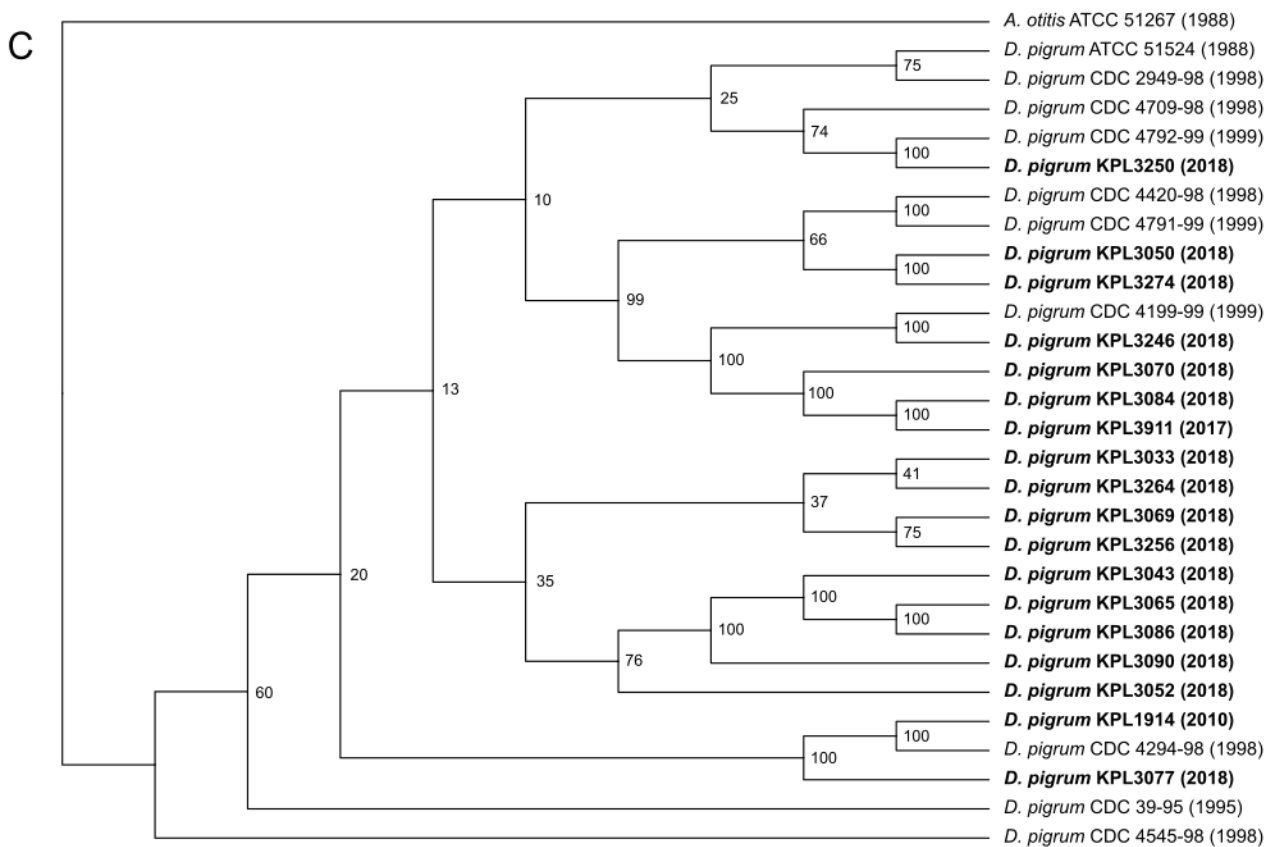

Supplement: FIG S1 [file msystems.00425-21-sf001.pdf]

A

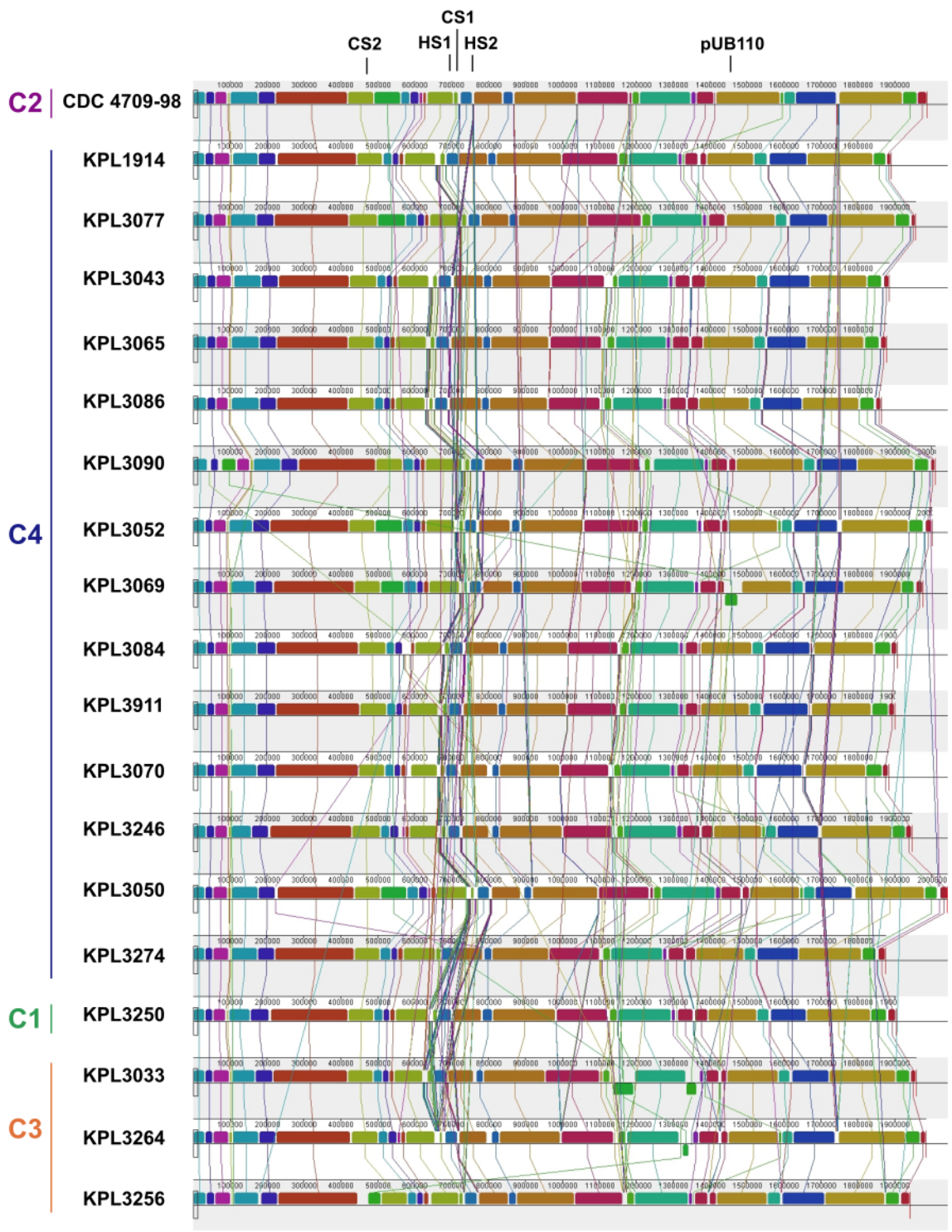

B

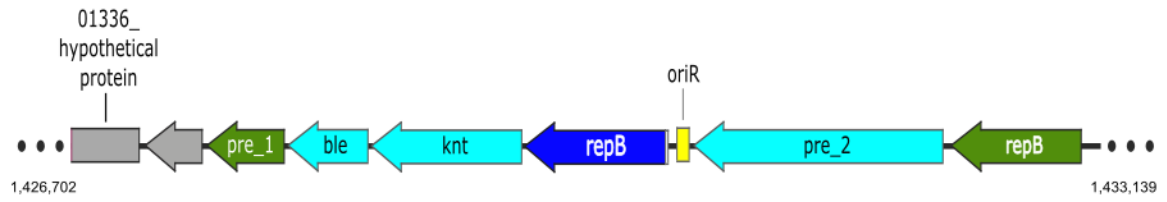

Supplement: FIG S2 [file msystems.00425-21-sf002.pdf]
