# Supplementary material for: Genomic Stability and Genetic Defense Systems in Dolosigranulum pigrum, a Candidate Beneficial Bacterium from the Human Microbiome
Source: mSystems. 2021 Sep 21;6(5):e00425-21. doi: 10.1128/mSystems.00425-21 (PMC8547433; doi:10.1128/mSystems.00425-21)
Supplement: FIG S3 [file msystems.00425-21-sf003.pdf]

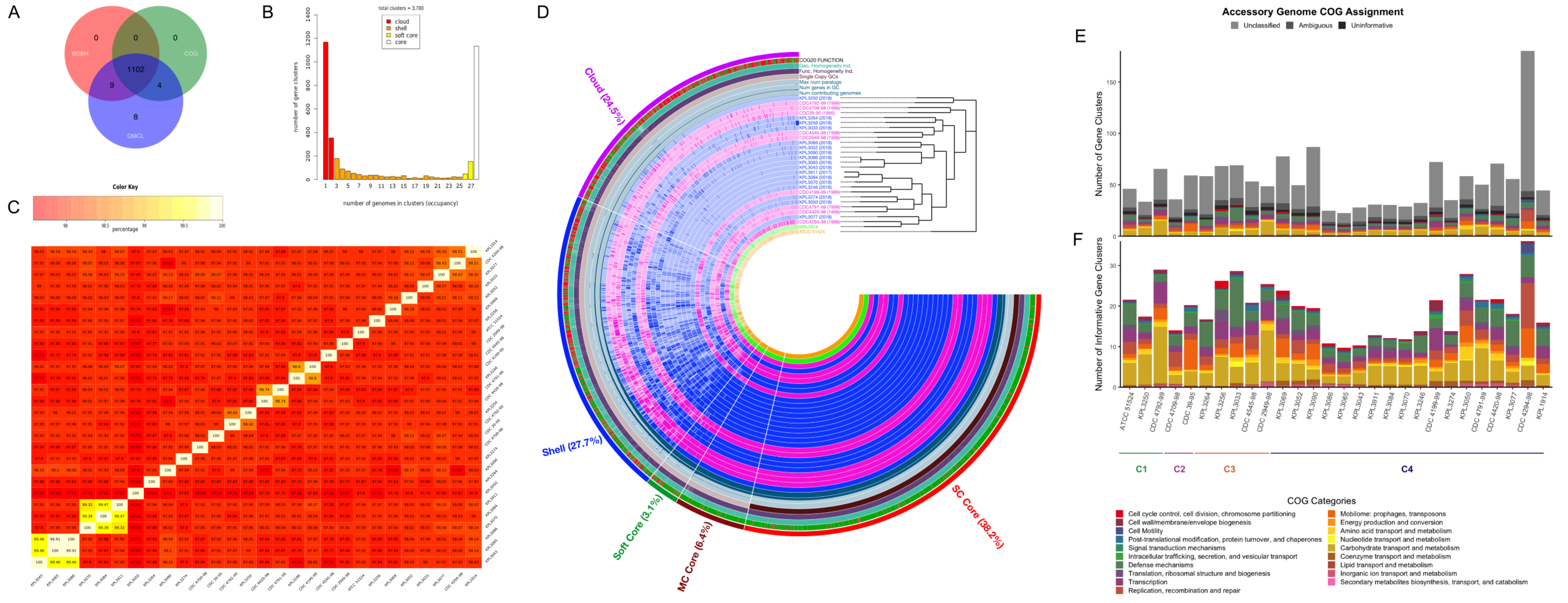
A
BDBH
COG
OMCL
0
0
0
1102
9
4
8
B
total clusters = 3,700
cloud
shell
soft core
core
number of gene clusters
number of genomes in clusters (occupancy)
C
Color Key
percentage
D
COG20 FUNCTION
Geo. Homogeneity Ind.
Func. Homogeneity Ind.
Single Copy GCs
Max num paralogs
Num genes in GC
Num contributing genomes
Cloud (24.5%)
Shell (27.7%)
Soft Core (3.1%)
MC Core (6.4%)
SC Core (38.2%)
Accessory Genome COG Assignment
Unclassified
Ambiguous
Uninformative
E
Number of Gene Clusters
F
Number of Informative Gene Clusters
C1
C2
C3
C4
COG Categories
Cell cycle control, cell division, chromosome partitioning
Cell wall/membrane/envelope biogenesis
Cell Motility
Post-translational modification, protein turnover, and chaperones
Signal transduction mechanisms
Intracellular trafficking, secretion, and vesicular transport
Defense mechanisms
Translation, ribosomal structure and biogenesis
Transcription
Replication, recombination and repair
Mobilome: prophages, transposons
Energy production and conversion
Amino acid transport and metabolism
Nucleotide transport and metabolism
Carbohydrate transport and metabolism
Coenzyme transport and metabolism
Lipid transport and metabolism
Inorganic ion transport and metabolism
Secondary metabolites biosynthesis, transport, and catabolism
