# Supplementary material for: Genomic Stability and Genetic Defense Systems in Dolosigranulum pigrum, a Candidate Beneficial Bacterium from the Human Microbiome
Source: mSystems. 2021 Sep 21;6(5):e00425-21. doi: 10.1128/mSystems.00425-21 (PMC8547433; doi:10.1128/mSystems.00425-21)
Supplement: TEXT S1 [file msystems.00425-21-s0001.pdf]

Text S1. Supplemental Analysis of Genetic Elements and Defense Systems

The four predicted *D. pigrum* prophage have few and dissimilar matches to known phages. We found four predicted mostly intact prophages in three of the *D. pigrum* genomes (**Fig. 5**) as defined by PHASTER's scoring system (Intact: score > 90; Questionable: score 70-90; Incomplete: score < 70): L1 in KPL3069 (score of 150; C4); L4 in KPL3256 (score 130; C3); and L2 and L3 in KPL3090 (score 130 and 100, respectively; C4). CDS from prophages L1-L4 displayed few and disparate matches to known phage genes. For example, prophage L1 had similarity to up to 30 different phage species among 56.3% (40/71) of its non-hypothetical proteins. The most common of these phage elements in L1 each only covered 11.3% (8/71) of the CDS in the phage region and were from a *Streptococcus pyogenes* host: temperate phage phiNIH1.1 (NC_003157) (1), and *Streptococcus* prophage 315.4 (NC_004587) (2). Temperate phage phiNIH1.1, which is integrated in the *Streptococcus pyogenes* M3/T3/subtype emm3.1 genome, is known for infecting lactic acid bacteria (1). Similarly, L4's most common match to known phages only had a 9.3% (7/75) CDS identity match to the temperate *Enterococcus* phage EFC-1 from *Enterococcus faecalis* KBL101 (NC_025453) (3). The most common phage hits for L2 and L3 were to *Streptococcus* phage phi O1205 (NC_004303) (4) and *Streptococcus* phage SMP (NC_008721) (5) with a 7.6% (6/79) and 15.8% (12/76) CDS percent identity, respectively. *Streptococcus* phage SMP is integrated in the genome of a *Streptococcus suis* type 2 strain, which was isolated from the nasal swab of a healthy Bama pig (5).

A subset of *D. pigrum* isolates encode for innate antibiotic resistance to kanamycin and/or erythromycin. Harmless members of human microbiota can serve as reservoirs of antibiotic resistance. Therefore, we searched for antibiotic resistance genes in these 28 *D. pigrum* genomes finding that six of the isolates encode predicted antibiotic resistance genes. Prediction of antibiotic resistance was determined by querying the genomes in parallel through the Comprehensive Antibiotic Resistance Database (CARD) in the Resistance Gene Identifier (RGI, version 3.1.0) API platform using default settings (6, 7). Results were considered significant if either a perfect or strict match based on a protein homolog AMR detection model was detected. Four genomes had a 100% identity match based on the RGI (6, 7) to a kanamycin nucleotidyltransferase ANT(4')-Ib that is encoded in integrated sequence homologous to pUB110 (CDC 4709-98, KPL3043, and KPL3065/KPL3086, the latter two having nearly identical genomes) (**Fig. S2B**). The latter three of these along with another clade 4 isolate (KPL3050) and a clade 1 isolate (KPL3250) also encoded an rRNA adenine N-6-methyltransferase (ErmT). With a strict identity match in the RGI of 86.53%, the predicted *ermT* gene is located within the CRISPR array of a subtype II-A system (**Fig. 8A-B**) in a different location in the genome than the kanamycin nucleotidyltransferase (CS1 vs. pUB110 in **Fig. S2A**).

Detailed description of *D. pigrum* restriction-modification (RM) systems. We identified Type I-IV RM systems across the 28 *D. pigrum* genomes located in three RMS insertion sites (**Fig. 7**)**.** Type I RM systems typically consist of three separate genes encoding a restriction subunit (*hsdR*), a modification subunit (*hsdM*) and a recognition/specificity (*hsdS*) subunit. These three form a multi-subunit complex to catalyze both restriction and modification activities that generally target bipartite DNA

motifs comprising two half-sequences separated by a gap (8). Two *D. pigrum* isolates, KPL3246 and KPL3264, were found to contain individual Type I systems and as each methylome contained characteristic bipartite motifs, CRTAN$_7$TCNNC and CTAN$_7$TGC respectively, associated with m6A modifications they were assigned as active.

Type II RM systems generally consist of independent restriction endonuclease (REase) and modification methyltransferase (MTase) proteins that do not form a complex, but instead recognize the same target motif and compete for activity. We identified 20 individual Type II systems across the 28 *D. pigrum* isolates and assigned the target motif to 15 of these based upon methylome analysis and/or REBASE homology to empirically characterized RM systems from other species (**Fig. 7A, Table S4**). A candidate motif was not detected for an m5C-associated RM system that co-occurred immediately downstream of the G^{m5}CNGC system in four isolates (KPL3264, KPL3911, KPL3084, and KPL3070). Additionally, we were unable to unambiguously assign motifs to two Type IIG RM systems that occurred in KPL3256 (REBASE assignments: Dpi3256ORF5220 and Dpi3256ORF1810), although well-informed guesses could be made (CCAGT and GACAG, respectively). Type IIG systems are defined by the presence of a single polypeptide including an REase and an MTase domain that share a target recognition domain. A single candidate modified motif for one of these systems was identified during SMRTseq (CC^{m6}AGT).

Type III RM systems consist of two genes (mod and res) encoding protein subunits that function either in DNA recognition and modification (MTase) or restriction (REase) activities. All Type III REases recognize asymmetrical DNA sequences and the modified

DNA bears methyl groups on only one strand of the DNA recognition motif. We found three *D. pigrum* isolates (KPL3274, KPL3052 and KPL3070) harbored characteristic Type III systems and assigned these to specific hemi-methylated recognition motifs (CAACA, GTCAT, YACAG) detected during methylome analysis (**Table S4**). In such systems, the REase must interact with two copies of its nonpalindromic recognition sequence and the sites must be in an inverse orientation within the substrate DNA molecule for cleavage, which occurs at a specific distance away from one of the recognition sequences.

Type IV restriction enzymes are technically not true RM systems since they comprise only a restriction enzyme and have no accompanying methylase. These restriction enzymes recognize and cut only modified DNA, including methylated, hydroxymethylated and glucosyl-hydroxymethylated bases. We identified a largely conserved single gene Type IV system in 10 *D. pigrum* isolates (**Fig. 7; Table S4**). However, the targets of Type IV systems cannot be determined through SMRT sequencing and methylome analysis and, therefore, exact determination of its target recognition motif was not possible here. Nevertheless, there are three well characterized Type IV systems to date each with defined sequence preference and cleavage position (9). The *D. pigrum* Type IV system is most homologous (99% coverage, 42% identity) to the SauUSI of *Staphylococcus aureus*; a modified cytosine restriction system targeting $S^{5m}CNGS$ (where S is C or G), but this level of homology is insufficient to confirm the exact modified motif targeted in the *D. pigrum* system (10).

D. pigrum **CRISPR spacers have few matches to known MGEs.** Two short-branched terminal clades (KPL3070, KPL3084, KPL3911 and KPL3043, KPL3065, KPL3086)

shared the most spacers among all the isolates. However, only 9 of their spacers had significant (cut-off score ≥ 15) matches to known MGEs. The KPL3070-containing clade (**Fig. 8**) had spacers with similarity to *Prochlorococcus* phage P-HM2 (GU075905; spacer 123) (11), *Lactobacillus plantarum* bacteriophage phiJL-1 (AY236756; spacer 131) (12), *Citrobacter* phage Moon (KM236240; spacer 136) (13), *Vibrio* phage 1.033.O._10N.222.49.B8 (MG592417; spacer 137) (14), *Pseudomonas sp.* Leaf58 plasmid pBASL58 (NZ_CP032678.1; spacer 142) (15), and the *Cupriavidus metallidurans* CH34 megaplasmid (NC_007974.2; spacer 143) (16). The KPL3043-containing clade had spacers with similarity to *Enterococcus* phage 156 (LR031359; spacer 7) (17), *Pectobacterium* phage CBB (KU574722; spacer 11) (18), and the *Bacillus* phage Page (KF669655; spacer 22) (19). Besides these two sets of closely related isolates, other less closely related isolates also shared more than three spacers. KPL3090 and KPL3052 both further apart in Clade 4 shared 7 spacers, which included matches to *Enterobacteria* phage RB49 (AY343333; spacer 39) (20) and the JP555 plasmid pJFP55H from *Clostridium perfringens* JP55 strain (NZ_CP013043.1; spacer 47) (21). All spacers sequences with their genome matches can be found in **Table S5B**.

Characterization of Virulence Factors. We found no matches when searching for predicted virulence factors among the 28 *D. pigrum* genomes by comparing to those encoded by *S. aureus* and *Enterococcus* using the VirulenceFinder2.0 online software (https://cge.cbs.dtu.dk/services/VirulenceFinder/) on 12/13/2020 (22).

REFERENCES

1. Ikebe T, Wada A, Inagaki Y, Sugama K, Suzuki R, Tanaka D, Tamaru A, Fujinaga Y, Abe Y, Shimizu Y, Watanabe H, Working Group for Group ASiJ. 2002. Dissemination of the phage-associated novel superantigen gene *speL* in recent invasive and noninvasive *Streptococcus pyogenes* M3/T3 isolates in Japan. Infect Immun 70:3227-33.
2. Beres SB, Sylva GL, Barbian KD, Lei B, Hoff JS, Mammarella ND, Liu M-Y, Smoot JC, Porcella SF, Parkins LD, Campbell DS, Smith TM, McCormick JK, Leung DYM, Schlievert PM, Musser JM. 2002. Genome sequence of a serotype M3 strain of group A *Streptococcus*: Phage-encoded toxins, the high-virulence phenotype, and clone emergence. Proc Natl Acad Sci USA 99:10078.
3. Yoon BH, Chang HI. 2015. Genomic annotation for the temperate phage EFC-1, isolated from *Enterococcus faecalis* KBL101. Arch Virol 160:601-4.
4. Stanley E, Fitzgerald GF, Marrec CL, Fayard B, van Sinderen D. 1997. Sequence analysis and characterization of phi O1205, a temperate bacteriophage infecting *Streptococcus thermophilus* CNRZ1205. Microbiology (Reading) 143 (Pt 11):3417-3429.
5. Ma YL, Lu CP. 2008. Isolation and identification of a bacteriophage capable of infecting *Streptococcus suis* type 2 strains. Vet Microbiol 132:340-7.
6. McArthur AG, Waglechner N, Nizam F, Yan A, Azad MA, Baylay AJ, Bhullar K, Canova MJ, De Pascale G, Ejim L, Kalan L, King AM, Koteva K, Morar M, Mulvey MR, O'Brien JS, Pawlowski AC, Piddock LJ, Spanogiannopoulos P, Sutherland AD, Tang I, Taylor PL, Thaker M, Wang W, Yan M, Yu T, Wright GD. 2013. The

comprehensive antibiotic resistance database. Antimicrob Agents Chemother 57:3348-57.

7. Jia B, Raphenya AR, Alcock B, Waglechner N, Guo P, Tsang KK, Lago BA, Dave BM, Pereira S, Sharma AN, Doshi S, Courtot M, Lo R, Williams LE, Frye JG, Elsayegh T, Sardar D, Westman EL, Pawlowski AC, Johnson TA, Brinkman FS, Wright GD, McArthur AG. 2017. CARD 2017: expansion and model-centric curation of the comprehensive antibiotic resistance database. Nucleic Acids Res 45:D566-D573.

8. Liu YP, Tang Q, Zhang JZ, Tian LF, Gao P, Yan XX. 2017. Structural basis underlying complex assembly and conformational transition of the type I R-M system. Proc Natl Acad Sci U S A 114:11151-11156.

9. Roberts RJ, Vincze T, Posfai J, Macelis D. 2015. REBASE--a database for DNA restriction and modification: enzymes, genes and genomes. Nucleic Acids Res 43:D298-9.

10. Xu SY, Corvaglia AR, Chan SH, Zheng Y, Linder P. 2011. A type IV modification-dependent restriction enzyme SauUSI from *Staphylococcus aureus* subsp. *aureus* USA300. Nucleic Acids Res 39:5597-610.

11. Sullivan MB, Huang KH, Ignacio-Espinoza JC, Berlin AM, Kelly L, Weigele PR, DeFrancesco AS, Kern SE, Thompson LR, Young S, Yandava C, Fu R, Krastins B, Chase M, Sarracino D, Osburne MS, Henn MR, Chisholm SW. 2010. Genomic analysis of oceanic cyanobacterial myoviruses compared with T4-like myoviruses from diverse hosts and environments. Environ Microbiol 12:3035-56.

12. Lu Z, Breidt F, Jr., Fleming HP, Altermann E, Klaenhammer TR. 2003. Isolation and characterization of a *Lactobacillus plantarum* bacteriophage, phiJL-1, from a cucumber fermentation. Int J Food Microbiol 84:225-35.

13. Edwards GB, Luna AJ, Hernandez AC, Kuty Everett GF. 2015. Complete Genome Sequence of *Citrobacter freundii* Myophage Moon. Genome Announcements 3:e01427-14.

14. Kauffman KM, Brown JM, Sharma RS, VanInsberghe D, Elsherbini J, Polz M, Kelly L. 2018. Viruses of the Nahant Collection, characterization of 251 marine *Vibrionaceae* viruses. Sci Data 5:180114.

15. Smith BA, Leligdon C, Baltrus DA. 2019. Just the Two of Us? A Family of *Pseudomonas* Megaplasmids Offers a Rare Glimpse into the Evolution of Large Mobile Elements. Genome Biol Evol 11:1192-1206.

16. Janssen PJ, Van Houdt R, Moors H, Monsieurs P, Morin N, Michaux A, Benotmane MA, Leys N, Vallaeys T, Lapidus A, Monchy S, Médigue C, Taghavi S, McCorkle S, Dunn J, Van Der Lelie D, Mergeay M. 2010. The Complete Genome Sequence of *Cupriavidus metallidurans* Strain CH34, a Master Survivalist in Harsh and Anthropogenic Environments. PLoS ONE 5:e10433.

17. Del Rio B, Sanchez-Llana E, Redruello B, Magadan AH, Fernandez M, Martin MC, Ladero V, Alvarez MA. 2019. *Enterococcus faecalis* Bacteriophage 156 Is an Effective Biotechnological Tool for Reducing the Presence of Tyramine and Putrescine in an Experimental Cheese Model. Front Microbiol 10:566.

18. Buttimer C, Hendrix H, Oliveira H, Casey A, Neve H, McAuliffe O, Ross RP, Hill C, Noben JP, O'Mahony J, Lavigne R, Coffey A. 2017. Things Are Getting Hairy: Enterobacteria Bacteriophage vB_PcaM_CBB. Front Microbiol 8:44.

19. Lopez MS, Hodde MK, Chamakura KR, Kuty Everett GF. 2014. Complete Genome of *Bacillus megaterium* Podophage Page. Genome Announcements 2:e00332-14.

20. Monod C, Repoila F, Kutateladze M, Tetart F, Krisch HM. 1997. The genome of the pseudo T-even bacteriophages, a diverse group that resembles T4. J Mol Biol 267:237-49.

21. Mehdizadeh Gohari I, Kropinski AM, Weese SJ, Parreira VR, Whitehead AE, Boerlin P, Prescott JF. 2016. Plasmid Characterization and Chromosome Analysis of Two netF+ *Clostridium perfringens* Isolates Associated with Foal and Canine Necrotizing Enteritis. PLOS ONE 11:e0148344.

22. Joensen KG, Scheutz F, Lund O, Hasman H, Kaas RS, Nielsen EM, Aarestrup FM. 2014. Real-time whole-genome sequencing for routine typing, surveillance, and outbreak detection of verotoxigenic *Escherichia coli*. J Clin Microbiol 52:1501-10.
