# Supplementary material for: Genomic Stability and Genetic Defense Systems in Dolosigranulum pigrum, a Candidate Beneficial Bacterium from the Human Microbiome
Source: mSystems. 2021 Sep 21;6(5):e00425-21. doi: 10.1128/mSystems.00425-21 (PMC8547433; doi:10.1128/mSystems.00425-21)
Supplement: TABLE S3 [file msystems.00425-21-st003.pdf]

Table S3. Predicted *D. pigrum* transposases, integrases and group II intron.

GENOMES	TRANSPOSASES									INTEGRASES						INTRON
	GC_00000003	GC_00000040	GC_00000055	GC_00001693	GC_00002092	GC_00002210	GC_00002310	GC_00002501	Total	GC_00000028	GC_00000085	GC_00001701	GC_00001775	GC_00002348	Total	GC_00000001
ATCC_51524	3	1	1	1					6		1				1	1
KPL1914											1		1		2	2
KPL1922_CDC39_95	1								1							1
KPL1930_CDC2949_98										1					1	2
KPL1931_CDC4294_98								1	1							2
KPL1932_CDC4420_98	2								2			1	1		2	3
KPL1933_CDC4545_98		1		1	1	1			4	2					2	1
KPL1934_CDC4709_98	6			1					7		1	1			2	11
KPL1937_CDC4199_99	2	1	1						4							2
KPL1938_CDC4791_99	1			1					2		1				1	1
KPL1939_CDC4792_99		1			1	1			3	1					1	2
KPL3033	1								1		1				1	5
KPL3043	1								1	1					1	9
KPL3050	4			1					5	2	1	3			6	10
KPL3052	1						1		2		1	1		1	3	4
KPL3065	1								1	1					1	7
KPL3069	1								1	1		1			2	2
KPL3070	5	1	1	1					8		1				1	5
KPL3077	11	1	1						13	1			1		2	2
KPL3084	7	2	2	1					12		1				1	6
KPL3086	1								1							6
KPL3090	6			1					7	1	1	1			3	14
KPL3246	3								3							8
KPL3250	2	1			1	1			5	1	1				2	9
KPL3256	1	1	1	1					4							3
KPL3264	6			1					7		1				1	5
KPL3274							1		1	5				1	6	1
KPL3911	8	2	2	1					13		1				1	8
**Total**	74	12	9	11	3	3	2	1	115	17	13	8	3	2	43	132
**Mean**	3.36	1.2	1.29	1	1	1	1	1	4.42	1.55	1	1.33	1	1	1.95	4.71
**Median**	2	1	1	1	1	1	1	1	3.5	1	1	1	1	1	1.5	3.5
**Variance**	8.24	0.18	0.24	0	0	0	0		14.25	1.47	0	0.67	0	0	2.14	12.9
**SD**	2.87	0.42	0.49	0	0	0	0		3.78	1.21	0	0.82	0	0	1.46	3.59
**MAD**	1.48	0	0	0	0	0	0	0	3.71	0	0	0	0	0	0.74	3.71
**Min**	1	1	1	1	1	1	1	1	1	1	1	1	1	1	1	1
**Max**	11	2	2	1	1	1	1	1	13	5	1	3	1	1	6	14
