# Supplementary material for: Genomic Stability and Genetic Defense Systems in Dolosigranulum pigrum, a Candidate Beneficial Bacterium from the Human Microbiome
Source: mSystems. 2021 Sep 21;6(5):e00425-21. doi: 10.1128/mSystems.00425-21 (PMC8547433; doi:10.1128/mSystems.00425-21)
Supplement: FIG S4 [file msystems.00425-21-sf004.pdf]

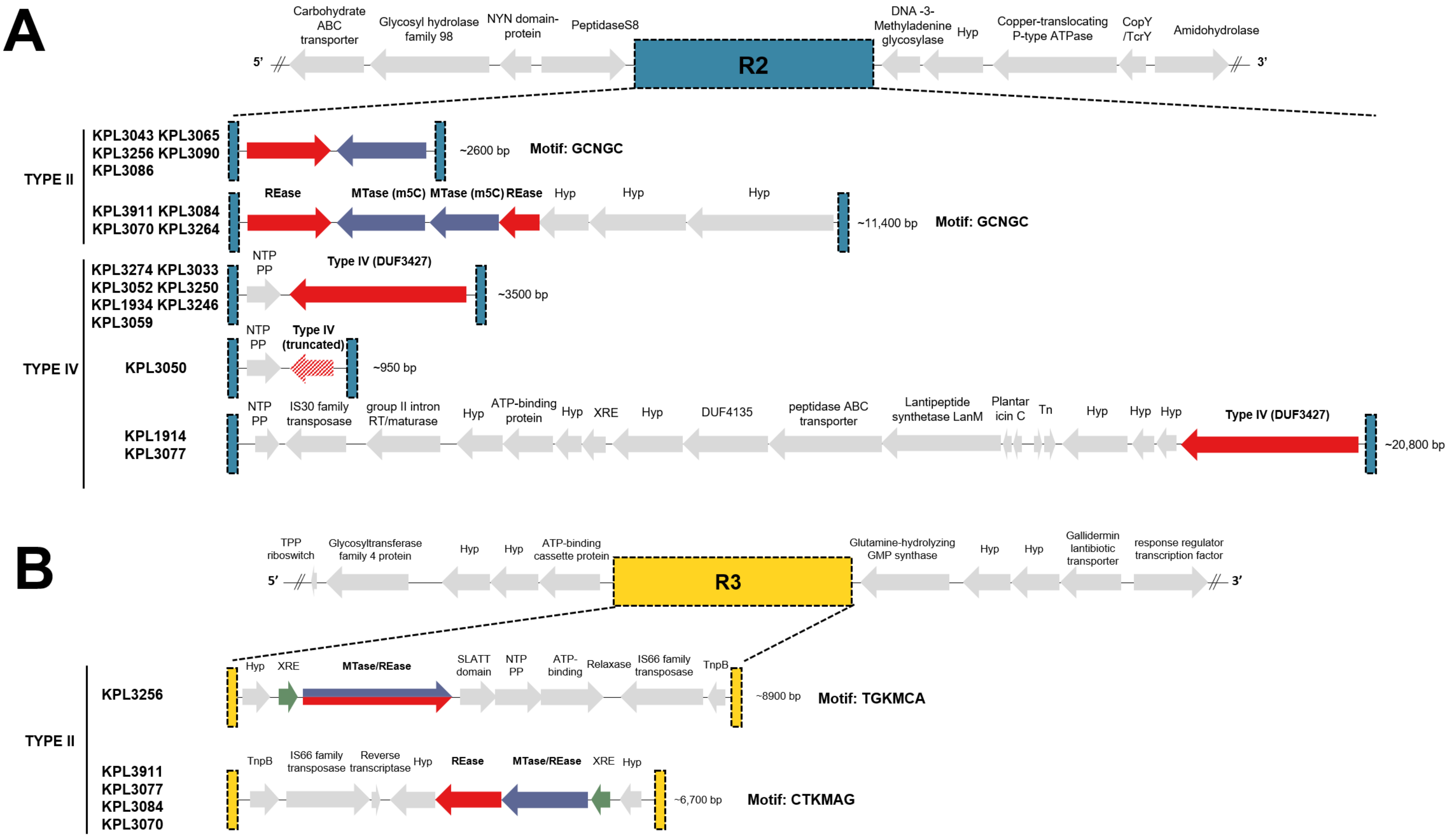
A
Carbohydrate ABC transporter
Glycosyl hydrolase family 98
NYN domain-protein
PeptidaseS8
R2
DNA -3-Methyladenine glycosylase
Hyp
Copper-translocating P-type ATPase
CopY /TcrY
Amidohydrolase
5'
3'
TYPE II
KPL3043 KPL3065 KPL3256 KPL3090 KPL3086
~2600 bp
Motif: GCNGC
REase
MTase (m5C)
MTase (m5C)
REase
Hyp
Hyp
Hyp
KPL3911 KPL3084 KPL3070 KPL3264
~11,400 bp
Motif: GCNGC
TYPE IV
KPL3274 KPL3033 KPL3052 KPL3250 KPL1934 KPL3246 KPL3059
NTP PP
Type IV (DUF3427)
~3500 bp
KPL3050
NTP PP
Type IV (truncated)
~950 bp
KPL1914 KPL3077
NTP PP
IS30 family transposase
group II intron RT/maturase
Hyp
ATP-binding protein
Hyp
XRE
Hyp
DUF4135
peptidase ABC transporter
Lantipeptide synthetase LanM
Plantar icin C
Tn
Hyp
Hyp
Hyp
Type IV (DUF3427)
~20,800 bp
B
TPP riboswitch
Glycosyltransferase family 4 protein
Hyp
Hyp
ATP-binding cassette protein
R3
Glutamine-hydrolyzing GMP synthase
Hyp
Hyp
Gallidermin lantibiotic transporter
response regulator transcription factor
5'
3'
TYPE II
KPL3256
Hyp
XRE
MTase/REase
SLATT domain
NTP PP
ATP-binding
Relaxase
IS66 family transposase
TnpB
~8900 bp
Motif: TGKMCA
KPL3911 KPL3077 KPL3084 KPL3070
TnpB
IS66 family transposase
Reverse transcriptase
Hyp
REase
MTase/REase
XRE
Hyp
~6,700 bp
Motif: CTKMAG
